# More Trustworthy Prediction of Elastic Modulus of Recycled Aggregate Concrete Using MCBE and TabPFN

**DOI:** 10.3390/ma18225221

**Published:** 2025-11-18

**Authors:** Wei-Tian Lu, Ze-Zhao Wang, Xin-Yu Zhao

**Affiliations:** State Key Laboratory of Subtropical Building and Urban Science, South China University of Technology, Guangzhou 510641, China; weitianlu1998@gmail.com (W.-T.L.);

**Keywords:** recycled aggregate concrete, elastic modulus, TabPFN, data bias, machine learning

## Abstract

The sustainable use of recycled aggregate concrete (RAC) is a critical pathway toward resource-efficient and environmentally responsible construction. However, the mechanical performance of RAC—particularly its elastic modulus—exhibits pronounced variability due to the heterogeneous quality and microstructural defects of recycled aggregates. This variability complicates the establishment of reliable predictive models and equations for elastic modulus estimation and restricts RAC’s broader structural implementation. Conventional empirical and machine-learning-based models (e.g., support vector machine, random forest, and artificial neural networks) are typically dataset-specific, prone to overfitting, and incapable of quantifying bias and uncertainty, making them unsuitable for heterogeneous materials data. This study introduces a bias-aware and more accurate predictive framework that integrates the Tabular Prior-data Fitted Network (TabPFN) with Monte Carlo Bias Estimation (MCBE)—for the first time applied in RAC materials research. A database containing 1161 RAC samples from diverse literature sources was established. This database includes key parameters such as apparent density ranging from 2270 kg/m^3^ to 3150 kg/m^3^, water absorption from 0.75% to 7.81%, replacement ratio from 0% to 100%, and compressive strength values ranging from 10.00 MPa to 108.51 MPa. MCBE quantified representational bias and guided targeted data augmentation, while TabPFN—pretrained on millions of Bayesian inference tasks—achieved *R*^2^ = 0.912 and RMSE = 1.65 GPa without any hyperparameter tuning. Feature attribution analysis confirmed compressive strength as the most influential factor governing the elastic modulus, consistent with established composite mechanics principles. The proposed TabPFN–MCBE framework provides a reliable, bias-corrected, and transferable approach for modeling recycled aggregate concrete (RAC). It enables accurate predictions that are both trustworthy and interpretable, advancing the use of data-driven methods in sustainable materials design.

## 1. Introduction

The exponential expansion of the global construction sector has drastically intensified the generation of construction and demolition (C&D) waste, which now accounts for up to 40% of urban solid waste in many metropolitan areas [1,2,3,4,5]. The inert yet voluminous nature of concrete debris poses severe challenges to landfill capacity and environmental sustainability [6]. At the same time, the extraction of natural aggregates (NA)—the primary constituent of concrete—is consuming high-quality reserves at an alarming rate [7,8]. These dual pressures of resource depletion and waste accumulation have accelerated the global transition toward a circular construction economy, in which waste materials are reintroduced into the production cycle.

Consequently, recycling demolished concrete into recycled aggregate (RA) has been increasingly promoted through both regulatory initiatives and engineering practices [9,10,11,12,13]. Within this framework, the production and utilization of RA have gained increasing significance. Recycled aggregate concrete (RAC), produced by partially or fully replacing natural aggregate with RA, has been widely acknowledged as a technically viable and environmentally sustainable alternative to natural aggregate concrete (NAC). The rapid development of RAC research over the past three decades has established its fundamental feasibility, while also revealing persistent challenges associated with mechanical performance and long-term durability [14,15,16,17,18,19,20,21,22,23].

Among the various engineering properties, mechanical characteristics are of particular importance, as they directly determine the stiffness, strength, and service life of structural members [24,25,26,27,28,29,30,31,32,33]. Experimental studies consistently show that increasing the replacement ratio (*RR*) of RA leads to a decline in compressive, tensile, and flexural strength [26,27,34,35,36]. This degradation is primarily attributed to the adhered old mortar, which increases water absorption, reduces density, and introduces microcracks at the interfacial transition zone (ITZ) [36,37,38]. Typically, tensile and flexural strengths decrease by 6–20% compared with NAC [32,39,40], indicating that the microstructural weakness of RA systematically propagates into macroscopic mechanical deterioration.

The elastic modulus (*E*_c_)—which governs the stiffness, deflection, and dynamic response of structural members—shows a far more complex dependence on RA incorporation than strength parameters. Experimental studies consistently report lower *E*_c_ values for RAC compared with NAC, yet the magnitude of reduction varies substantially across studies [41,42]. Reported declines range from 12% to 38% at full replacement of recycled coarse aggregate [12,21,27,35], reflecting the influence of multiple interacting factors including RA replacement ratio, aggregate quality, and the heterogeneity of the ITZ between old and new mortar.

A closer examination of existing data reveals a tiered relationship between RA quality and *E*_c_ degradation. High-quality RA, derived from sound parent concrete and subjected to adequate processing, typically reduces *E*_c_ by only 3–17%. Medium- and low-quality RA, however, lead to reductions of approximately 10–26%, while non-standard aggregates can lower stiffness by up to 55% [43,44,45,46,47,48,49,50,51]. This stratified trend highlights that RA quality—not merely replacement ratio—plays an important role in determining stiffness retention. Enhancing aggregate quality through source selection, improved crushing, and mortar removal can therefore markedly mitigate the stiffness loss of RAC.

Despite these observations, the predictive understanding of *E*_c_ remains fragmented and inconsistent. While a statistically significant correlation between *E*_c_ and compressive strength (*f*_c_) has been widely reported [41], the relationship is highly dataset-dependent, sensitive to local testing methods, and biased by the uneven representation of aggregate quality levels. Consequently, existing empirical or regression-based correlations fail to generalize across studies, revealing the pressing need for more robust predictive models capable of accounting for data heterogeneity and bias in RAC datasets.

Indeed, no universal analytical relationship currently exists for predicting the elastic modulus of RAC. Classical code-based formulas are fundamentally empirical and were derived from datasets of NAC. When applied to RAC, these equations systematically overestimate *E*_c_, as they neglect the effects of adhered mortar, increased porosity, and the altered stiffness of the composite aggregate skeleton. Attempts to modify these expressions by introducing correction factors for RA replacement ratio or density have improved local accuracy but failed to generalize across datasets collected under different experimental conditions.

In view of these limitations, researchers have increasingly turned to data-driven modeling to capture the nonlinear and coupled influences of mixture design and aggregate characteristics. Machine learning (ML) techniques—such as Artificial Neural Networks (ANNs), Random Forests (RFs), and Support Vector Machines (SVMs)—have been widely employed to predict RAC properties, including compressive strength, tensile capacity, and elastic modulus [52,53,54,55]. These methods offer higher flexibility than empirical correlations and can uncover hidden patterns within multi-source data.

However, despite their promise, existing ML models still face three critical challenges. First, RAC datasets are inherently heterogeneous and biased, originating from different laboratories, mixture designs, and quality grades of RA. Conventional ML algorithms tend to overfit to these fragmented datasets, resulting in poor cross-domain generalization. Second, the black-box nature of many models limits physical interpretability, which hinders their adoption in engineering design and code calibration. Third, most ML frameworks require iterative retraining and hyperparameter optimization, imposing high computational cost and restricting scalability. These challenges collectively reveal the need for a new predictive paradigm that combines accuracy, efficiency, and uncertainty quantification while being robust to dataset bias.

**Figure 1:**
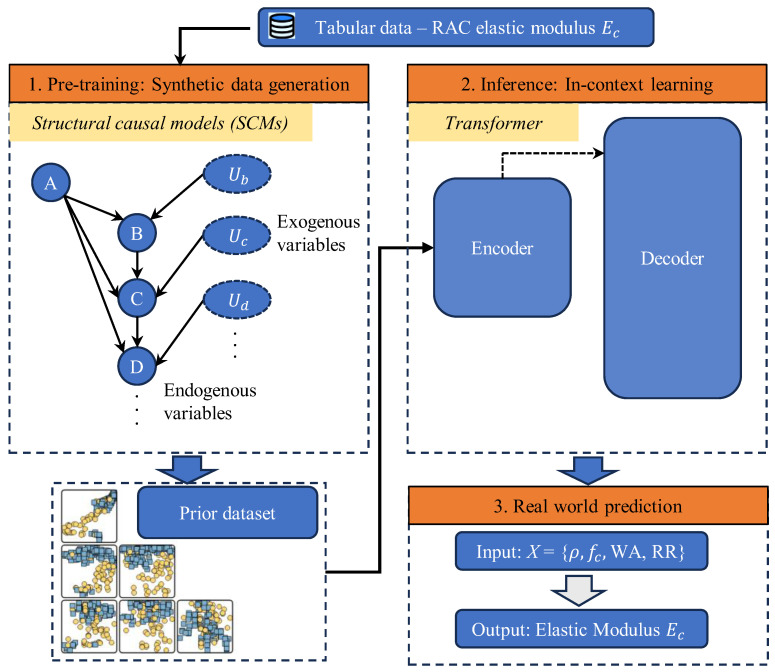
Principle and flowchart of TabPFN for predicting *E*_c_ of RAC.

In response to this need, the present study introduces the Tabular Prior-data Fitted Network (TabPFN)—a transformer-based foundation model originally developed for tabular inference—as a novel solution for predicting the elastic modulus (*E*_c_) of RAC [56,57,58,59]. The principle and workflow of TabPFN are illustrated in Figure 1. Unlike conventional machine learning algorithms that must be retrained for each dataset, TabPFN functions as a pretrained Bayesian learner, having been exposed to millions of synthetic tasks generated from Bayesian Neural Networks (BNNs) and Structural Causal Models (SCMs). This prior knowledge enables one-shot Bayesian prediction on small and heterogeneous datasets—precisely the regime in which most RAC databases exist.

Three properties make TabPFN particularly suited to this problem:

First, it inherently handles small and noisy datasets without overfitting, ensuring robust generalization across studies with different mixture designs, testing standards, and aggregate qualities.

Second, it directly approximates Bayesian inference, producing Posterior Predictive Distributions (PPDs) that quantify predictive uncertainty—an essential element for trustworthy, risk-informed design, where both overestimation and underestimation of *E*_c_ can lead to unsafe or uneconomical outcomes.

Third, it provides orders-of-magnitude faster inference than conventional AutoML pipelines, producing instant predictions through a single forward pass.

However, even the most advanced models remain constrained by the quality and representativeness of available data. Existing RAC datasets exhibit pronounced distributional imbalance and source bias, particularly in high-strength regions and varying aggregate qualities. To address this limitation, we integrate TabPFN with a Monte Carlo Bias Estimation (MCBE) module that quantifies and mitigates dataset bias through probabilistic reweighting and targeted data augmentation.

Hence, the novelty and contribution of this study are twofold:

(1) It represents the first integration of a foundation model (TabPFN) into the field of RAC mechanics, enabling knowledge transfer between data science and materials engineering; and
(2) It introduces a bias-aware data calibration framework (MCBE) that complements the model’s Bayesian learning with explicit bias quantification and correction.

Together, these advances establish a probabilistic, bias-aware, and trustworthy modeling framework that enhances accuracy, interpretability, and reliability in predicting the elastic modulus of RAC and lays the foundation for future performance-based and uncertainty-informed design of sustainable concrete materials.

## 2. Methodology

### 2.1. Tabular Prior-Data Fitted Network

TabPFN (Tabular Prior-Data Fitted Network) is a meta-learning framework that approximates Bayesian inference for supervised learning on tabular data by learning to predict the Posterior Predictive Distribution (PPD) from data. Given a training set $D_{train}={\left\{(x_{i},y_{i})\right\}}_{i=1}^{n}$ and a test (query) input *x*, the Bayesian PPD for its label *y* integrates over a hypothesis space *Φ*:

(1) $$p\left(y\;|\;x,\;D\right)\;\propto \;\int_{\phi \in \Phi }p\left(y\;|\;x,\;\phi \right)\;p\left(D_{train}\;|\;\phi \right)\;p\left(\phi \right)\;d\phi$$

where $p\left(y\;\right|\;x,\;\phi )$ is the likelihood of label *y* at input *x* under hypothesis ϕ; $p\left(D_{train}\;\right|\;\phi )$ is the marginal likelihood of the observed training data under ϕ; and p(ϕ) is the prior over hypotheses. The integral yields the PPD $p\left(y\;\right|\;x,\;D_{train})$. Directly computing (1) is generally intractable for rich priors and datasets, motivating a learned approximation.

TabPFN instantiates the general idea of a Prior-Data Fitted Network (PFN) by training a parametric model $q_{\theta }\left(y\;\right|\;x,\;D_{train})$ to approximate the Bayesian PPD for datasets sampled from a prior over tasks. Concretely, the prior is specified by sampling scheme $p\left(D\right)=E_{\phi \sim p\left(\phi \right)}[p\left(D\;\right|\;\phi )]$: first sample a data-generating mechanism *ϕ*, then sample a dataset *D* from it. In TabPFN the mechanism prior is mixed from two complementary families. A Bayesian Neural Network (BNN) prior draws neural architectures and weights (and noise) and generates supervised pairs by forward-propagating inputs, thereby capturing complex, nonlinear dependencies; a Structural Causal Model (SCM) prior draws a DAG and structural assignments $z_{i}=\;f_{i}(z_{pa\left(i\right)},\varepsilon_{i})$, then designates observed nodes $z_{x}$ as features and $z_{y}$ as the label to synthesize tabular datasets that reflect causal feature-label relations. Mixing BNN and SCM priors yields a broad, human-plausible hypothesis space that TabPFN can learn to marginalize over.

Formally, TabPFN fits $q_{\theta }$ by minimizing expected cross-entropy on held-out test points drawn from the prior. For a dataset split into $D_{train}$ and a single held-out pair $D_{test}=\{(x,\;y)\}$, the loss is:

(2) $$L_{PFN}=E_{\left\{\left(x,\;y\right)\right\}\cup D_{train}\;p\left(D\right)}\left[-\log q_{\theta }\left(y\;|\;x,\;D_{train}\right)\right]$$

where $q_{\theta }\left(y\;\right|\;x,\;D_{train})$ is the PFN’s approximate PPD; minimizing this expectation (with D ~ p(D)) provably drives $q_{\theta }$ toward the true Bayesian PPD under the chosen prior. This synthetic prior-fitting is performed once, offline, producing a single network reusable across target datasets.

Implementation-wise, TabPFN realizes $q_{\theta }$ with a Transformer that tokenizes each training pair $\left(x_{i},\;y_{i}\right)$ and each query *x*; attention masks enforce that training tokens attend to one another while query tokens attend only to training tokens, thereby computing $q_{\theta }\left(\cdot \right|\;x,\;D_{train})$ in one forward pass without any task-specific gradient updates.

It is noteworthy that in our actual code implementation, the SCM component of TabPFN is employed to generate synthetic datasets that conform to causal relationships. Specifically, we define apparent density (*ρ*), water absorption (*WA*), replacement ratio (*RR*), and compressive strength (*f*_c_) as exogenous variables, while elastic modulus (*E*_c_) serves as the output variable. This configuration reflects the physical causal relationships inherent in RAC: apparent density, water absorption, replacement ratio, and compressive strength, as fundamental material characteristics and performance indicators, collectively determine the magnitude of the elastic modulus. In the Directed Acyclic Graph (DAG) of the SCM, these four exogenous variables all point to *E*_c_, indicating their direct causal influence on the elastic modulus, thereby forming a simplified causal structure.

It should be noted that to simplify the model and focus on the primary influencing factors, this study does not incorporate endogenous variables. Future research could introduce endogenous variables to construct more complex causal networks, thereby more comprehensively capturing the interdependencies among RAC material properties and further enhancing both predictive accuracy and physical interpretability of the model.

**Figure 2:**
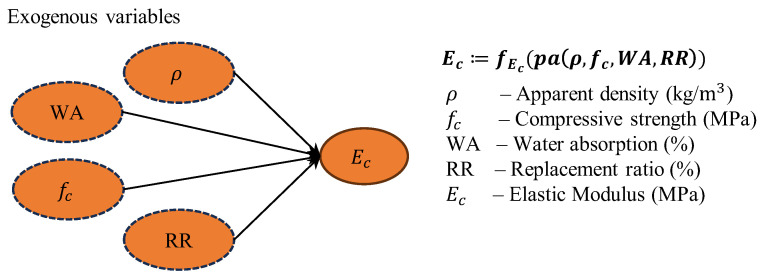
Exogenous variables for predicting *E*_c_ of RAC.

Through this explicit definition of causal structure, TabPFN learns prior knowledge consistent with the mechanical principles of RAC materials during the pre-training phase, thus demonstrating superior generalization capability when confronted with actual data. This causal relationship diagram is illustrated in Figure 2, which clearly displays the dependency structure between input features and the target variable.

### 2.2. Artificial Neural Networks

Artificial Neural Networks (ANNs) are computational models inspired by biological neural systems. Their basic structure consists of multiple artificial neurons connected through weighted links to form a network. Each neuron receives input signals, sums them, and generates an output through an activation function. This process simulates how biological neurons process signals. The advantage of ANNs lies in their powerful pattern recognition and learning abilities, especially in handling complex nonlinear relationships.

An ANN typically consists of an input layer, hidden layers, and an output layer. The input layer receives data, the output layer generates prediction results, and the hidden layers are responsible for processing information. Each neuron exchanges information with other neurons through weights, which are adjustable parameters. The computation in an ANN is determined by the weighted sum of the input signals and the activation function.

Mathematically, the net input for a neuron is the weighted sum of the input signals:

(3) $$NET_{j}\;=\;\sum_{i=1}^{m}w_{ji}x_{i}\;+\;b_{j}$$

where $w_{ji}$ is the weight between the input signal $x_{i}$ and the neuron, determining the extent to which the input signal influences the output; $x_{i}$ is the *i*-th input signal, representing the external data received by the neuron; and $b_{j}$ is the bias term, which ensures that the neuron’s output can adapt to different data distributions. The net input is passed through an activation function to produce the output signal, enabling the ANN to capture complex relationships between the input signals.

### 2.3. Random Forest

Random Forest (RF) is an ensemble learning method widely used for classification and regression tasks. It builds multiple decision trees using random subsets of data, and aggregates their predictions to improve accuracy and robustness. Each tree in the forest is trained on a bootstrap sample drawn from the original dataset, and at each split, a random subset of features is selected, making the model resistant to overfitting.

The general Random Forest model is formulated as follows: for a dataset with feature vectors $X=\{x_{1},x_{2},\ldots ,x_{n}\}$ and target variables $Y=\{y_{1},y_{2},\ldots ,y_{n}\}$, each tree *t* is trained on a random bootstrap sample and uses a random subset of features at each node. The final prediction $\hat{f}(x)$ is the average of predictions from all trees:

(4) $$\hat{f}\left(x\right)=\;\frac{1}{T}\sum_{t=1}^{T}h_{t}\left(x\right)$$

where $h_{t}(x)$ is the prediction from the *t*-th tree; and *T* is the total number of trees. The strength of a Random Forest comes from both the random sampling of data and features, and the aggregation of predictions, which helps reduce variance and avoid overfitting. As more trees are added, the model’s generalization error tends to converge, and the overall prediction becomes more stable. The forest’s performance is influenced by the strength of individual trees and the correlation between them, which are controlled through randomization at each step.

Additionally, Random Forests provide internal measures of feature importance by evaluating how much the accuracy decreases when a feature is permuted. This allows the model to identify which features contribute most to the prediction.

### 2.4. Extreme Gradient Boosting

XGBoost (eXtreme Gradient Boosting) is an efficient implementation of the gradient boosting framework that has become one of the most widely used machine learning algorithms in structured data domains due to its exceptional computational efficiency and predictive performance. Developed by Chen and Guestrin [60], this algorithm demonstrates outstanding performance across numerous application areas. The fundamental principle of XGBoost builds upon Gradient Boosted Decision Trees (GBDTs), where the model is constructed in an additive manner. Given a dataset with *n* examples and *m* features, XGBoost uses *K* additive functions to predict the output:

(5) $${\hat{y}}_{i}=\phi \left(x_{i}\right)=\sum_{k=1}^{K}f_{k}\left(x_{i}\right),\;\;f_{k}\;\in \;F$$

where ${\hat{y}}_{i}$ is the predicted value for the *i*-th instance; $\phi (x_{i})$ represents the ensemble model; $x_{i}$ denotes the feature vector of the *i*-th instance; *K* is the total number of trees in the ensemble; $f_{k}$ represents the *k*-th tree function; and F denotes the space of all possible regression trees (CART), with each tree structure mapping input features to leaf scores.

To learn this ensemble of functions, XGBoost minimizes a regularized objective function:

(6) $$L\left(\phi \right)=\;\sum_{i}l\left({\hat{y}}_{i},y_{i}\right)+\sum_{k}\Omega \left(f_{k}\right)$$

where $L\left(\phi \right)$ is the overall objective function to be minimized; $l({\hat{y}}_{i},y_{i})$ is a differentiable convex loss function that measures the difference between the predicted value ${\hat{y}}_{i}$ and the true label $y_{i}$; and $\Omega \left(f_{k}\right)\;=\;\gamma T\;+\;\frac{1}{2}\lambda \|w{\|}^{2}$ is the regularization term that penalizes model complexity, in which *T* denotes the number of leaves in tree, $f_{k}$ is the complexity cost per leaf, *λ* is the *L_2_* regularization parameter, and *w* represents the vector of scores on the leaves. This regularization term prevents overfitting by favoring simpler models.

The model is trained additively through gradient boosting. At iteration *t*, a new tree $f_{t}$ is added to minimize the objective function. Using second-order Taylor approximation, the objective can be rewritten as:

(7) $${\tilde{L}}^{\left(t\right)}=\;\sum_{i=1}^{n}\left[g_{i}f_{t}\left(x_{i}\right)+\frac{1}{2}h_{i}f_{t}^{2}\left(x_{i}\right)\right]+\Omega \left(f_{t}\right)$$

where ${\tilde{L}}^{\left(t\right)}$ is the simplified objective at iteration *t* after removing constant terms; *n* is the total number of training instances; $g_{i}\;=\;\partial_{{\hat{y}}^{\left(t-1\right)}}l\left(y_{i},{\hat{y}}^{\left(t-1\right)}\right)$ is the first-order gradient statistic (first derivative of the loss function with respect to the prediction at iteration *t* − 1); $h_{i}\;=\;\partial_{{\hat{y}}^{\left(t-1\right)}}^{2}l\left(y_{i},{\hat{y}}^{\left(t-1\right)}\right)$ is the second-order gradient statistic (second derivative of the loss function); $f_{t}(x_{i})$ is the prediction of the new tree being added at iteration *t*; and $\Omega \left(f_{t}\right)$ is the complexity penalty of the new tree. For a fixed tree structure, the optimal leaf weights and corresponding loss value can be computed analytically, enabling efficient split finding.

XGBoost incorporates several algorithmic and system-level optimizations that distinguish it from traditional implementations. These include a sparsity-aware split finding algorithm for handling missing values, weighted quantile sketch for approximate tree learning, column block structure for parallel computation, and support for both exact greedy algorithms and approximate algorithms with global or local proposals, providing flexibility for different dataset scales and computational constraints.

The practical advantages of XGBoost include: (1) superior computational speed through parallelization and cache-aware optimization; (2) built-in regularization to reduce overfitting; (3) efficient handling of sparse data and missing values; (4) support for custom objective functions and evaluation metrics; and (5) scalability to large datasets through out-of-core computation and distributed processing. These features have made XGBoost the algorithm of choice in numerous machine learning competitions and real-world applications, particularly in domains involving structured tabular data.

### 2.5. Gradient Boosted Decision Trees

Gradient Boosted Decision Trees (GBDTs) are an ensemble learning method that builds a strong predictive model by iteratively combining multiple decision trees. It works by sequentially adding trees, where each tree aims to correct the errors made by the previous one. The process is guided by gradient descent to minimize the loss function.

The model starts with an initial prediction $F_{0}\left(x\right)$, which is often set to the mean of the target variable. In each boosting iteration mmm, the model is updated as:

(8) $$F_{m}\left(x\right)=F_{m-1}\left(x\right)\;+\;\eta_{m}h_{m}\left(x\right)$$

where $F_{m}(x)$ is the prediction after the *m*-th iteration; $F_{m-1}(x)$ is the prediction after the previous iteration; $\eta_{m}$ is the learning rate that controls the contribution of the new tree to the model; and $h_{m}(x)$ is the decision tree added in the *m*-th iteration to correct the residuals of the previous model.

Each tree $h_{m}(x)$ is trained to fit the residuals, which are the errors of the current prediction. The residuals are calculated as the difference between the true value and the model’s current prediction. The tree is fitted by minimizing the following loss:

(9) $$h_{m}\left(x\right)=arg\;\underset{h}{\min}\sum_{i=1}^{N}{\left[y_{i}-F_{m-1}\left(x_{i}\right)-h\left(x_{i}\right)\right]}^{2}$$

where $y_{i}$ is the true value of the *i*-th sample; $F_{m-1}(x_{i})$ is the predicted value of the *i*-th sample from the previous model; and $h(x_{i})$ is the predicted value from the decision tree for the *i*-th sample.

Once the tree is constructed, the coefficient $\alpha_{m}$ is determined by minimizing the loss function:

(10) $$\alpha_{m}=arg\;\underset{\alpha }{\min}\sum_{i=1}^{N}{\left[y_{i}-F_{m-1}\left(x_{i}\right)-\alpha h_{m}\left(x_{i}\right)\right]}^{2}$$

where $\alpha_{m}$ is the coefficient for the new tree that adjusts its contribution to the overall prediction; and $h_{m}(x_{i})$ is the decision tree added in the *m*-th iteration.

Through this iterative process, each new tree is trained to reduce the error of the model, improving the overall predictive performance. GBDT is known for its accuracy and robustness, and enhancements like XGBoost and LightGBM improve both training speed and prediction accuracy.

### 2.6. Support Vector Machines

Support Vector Machines (SVMs) are powerful supervised learning algorithms primarily used for classification tasks. The goal of SVM is to find a hyperplane that separates data points into two classes with the largest possible margin. This separation ensures the best generalization to new, unseen data.

In its simplest form, SVM attempts to find a hyperplane in a high-dimensional space that separates the two classes. The equation for a hyperplane is given by:

(11) $$\begin{array}{c} w\cdot x+b=0 \end{array}$$

where *w* is the weight vector, determining the direction of the hyperplane; *x* represents the input data points; and *b* is the bias term, which shifts the hyperplane along the axis. The objective is to maximize the margin, which is the distance between the hyperplane and the nearest data points from each class. This is mathematically equivalent to minimizing the following objective function, which aims to reduce the size of the weight vector *w*:

(12) $$\frac{1}{2}\|w{\|}^{2}$$

In practical scenarios, the data may not always be perfectly separable. To handle such cases, SVM uses a “soft margin” approach, where some data points are allowed to be misclassified, and a regularization parameter *C* is introduced. The trade-off between a large margin and fewer misclassifications is controlled by *C*. To solve this, SVM uses a kernel function, allowing for nonlinear decision boundaries. The kernel function computes the inner product between the data points in the higher-dimensional space without explicitly mapping them there, making the computations more efficient. Thus, SVM is a robust and flexible model that handles both linear and nonlinear classification tasks efficiently by finding the optimal decision boundary that maximizes the margin.

## 3. Experimental Database and Data Processing

A total of 1161 data points were compiled from a broad and diverse collection of published studies on RAC [19,28,31,36,37,46,47,48,50,61,62,63,64,65,66,67,68,69,70,71,72,73,74,75,76,77,78,79,80,81,82,83,84,85,86,87,88,89,90,91,92,93,94,95,96,97,98,99,100,101,102,103,104,105,106,107,108,109,110,111,112,113,114,115,116,117,118,119,120,121,122,123,124,125,126,127,128,129,130,131,132,133,134,135]. Previous databases for predicting the elastic modulus of RAC have incorporated a wide range of mixture and material parameters, including type of aggregate, water/cement ratio, age of test, transition zone, curing age and other material characteristics. However, from an engineering application perspective, this study prioritizes parameters that are more readily accessible and practical in real-world construction scenarios. Rather than focusing solely on detailed material composition, we emphasize parameters such as apparent density, water absorption, replacement ratio of recycled aggregate (RA), and compressive strength—all of which are commonly available in engineering practice. These parameters offer distinct advantages: the testing methods are well-established, cost-effective, and straightforward to implement. For instance, apparent density can be determined using standard ASTM methods, water absorption is typically measured through simple immersion tests, and compressive strength is routinely assessed via standard concrete compression tests. Additionally, if too many input features are concerned, such comprehensive datasets inevitably introduce issues with missing data, limiting the suitability for bias analysis.

In this study, the focus was placed on establishing a clean, harmonized, and bias-evaluable dataset rather than maximizing input parameters. Consequently, four fundamental and consistently reported parameters were retained as input features: apparent density, water absorption, RA replacement ratio, and compressive strength (*f*_c_). These variables collectively capture both the intrinsic material properties of the recycled aggregate and the mechanical response of the composite matrix, providing a concise yet physically meaningful basis for elastic modulus prediction. Moreover, the strong predictive performance using only these four variables confirms that they are the primary factors influencing RAC elastic modulus.

The data were drawn from multiple independent laboratories employing diverse testing standards and procedures. Such heterogeneity necessitated a comprehensive preprocessing stage prior to model development, which included (i) normalization of measurement units, (ii) reconciliation of specimen size effects, and (iii) removal of anomalous or inconsistent records. This ensured the comparability and reliability of the compiled dataset for subsequent bias quantification and model training.

### 3.1. Outlier Analyses

Given the heterogeneity of the compiled database—arising from differences in RA quality classes, specimen preparation procedures, and testing protocols across the various source studies—an additional round of outlier screening was performed to ensure statistical robustness prior to the TabPFN modeling.

Outlier detection was carried out using a Gaussian Mixture Model (GMM) [136,137]. The GMM is a generative probabilistic model that represents the underlying data distribution as a weighted combination of multiple Gaussian components. This formulation is conceptually aligned with Bayesian Neural Networks, which also treats parameters as random variables drawn from specified probability distributions. In the present context, GMM effectively identifies data points with a low posterior probability of belonging to any of the main data clusters, thereby flagging them as potential outliers.

**Figure 3:**
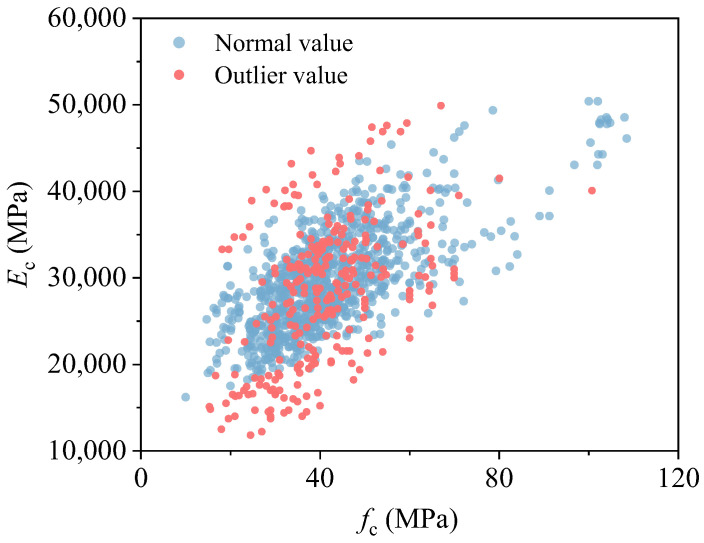
Outlier data identification.

Through this unified GMM-based procedure, a total of 195 samples—representing 16.8% of the dataset—were classified as outliers and removed from subsequent analysis. In the initial scatter plots of elastic modulus versus compressive strength, as shown in Figure 3, the majority of data points were concentrated at the peripheries, reflecting a scattered and unorganized data distribution.

Prior to outlier removal, the observed maximum variation in *E*_c_ for specimens with the same compressive strength exceeded 30,000 MPa, which is a highly unrealistic range for concrete materials. This extreme variation suggests the presence of significant errors or inconsistencies within the dataset.

After systematically removing these outliers, the data distribution became more compact, and the extreme values were significantly reduced. The cleaned dataset now exhibits a more reliable and consistent range of *E*_c_ for each strength level, which is more in line with the well-established mechanical behavior of RAC. These improvements in data consistency, ensure that the dataset is more representative of the actual material properties and are crucial for any subsequent analyses or modeling.

### 3.2. Dataset Characterization

**Figure 4:**
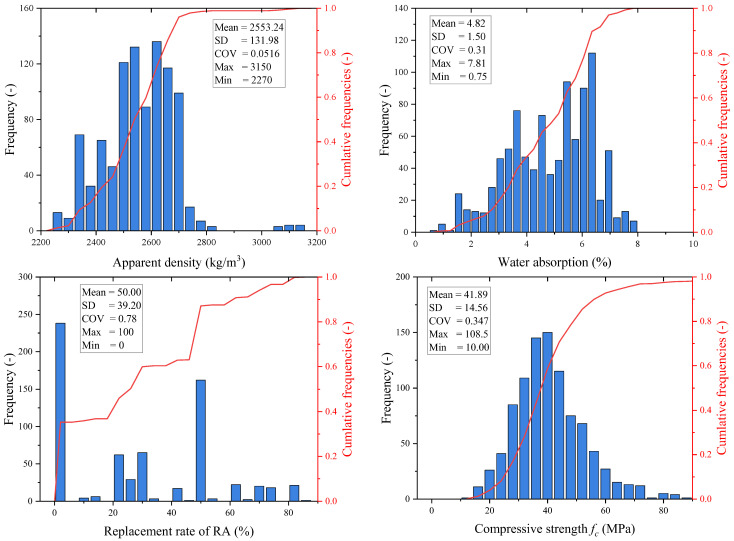
Distribution and basic statistical parameters of variables.

The distribution and basic statistical characteristic of the dataset variables are shown in Figure 4. The apparent density values follow an approximately normal distribution, with a mean of 2553.24 kg/m^3^ and a standard deviation (SD) of 131.98 kg/m^3^, yielding a coefficient of variation (COV) of 0.0516. The data range spans from 2270 kg/m^3^ to 3150 kg/m^3^, indicating a mix of lightweight and heavyweight concrete samples. However, the majority of the values concentrate between 2400 kg/m^3^ and 2800 kg/m^3^, with a notable peak in the 2500–2700 kg/m^3^ range, which is typical for concrete used in the study, making it a suitable feature for predictive modeling.

Water absorption values range from 0.75% to 7.81%, with a mean of 4.82% and an SD of 1.50%. The COV is 0.31, suggesting relatively low dispersion across the dataset. Approximately 60–70% of the samples show water absorption below 6%, indicating that most of the RAC samples exhibit good quality, though there is still considerable variability, particularly at higher absorption levels. This variability emphasizes the importance of water absorption in assessing the durability of RAC.

The replacement ratio of recycled aggregates presents a distinct bimodal distribution, with peaks at 0% and 100%, representing natural aggregate control and full replacement, respectively. The mean replacement rate is 50%, with a high SD of 39.20% and a COV of 0.78, reflecting the common experimental focus on these two extremes. This distribution pattern introduces challenges for modeling, as it may not fully represent intermediate replacement ratios. In terms of compressive strength, the values range from 10.00 MPa to 108.51 MPa, with a mean of 41.89 MPa, an SD of 14.56 MPa, and a COV of 0.347. Most samples fall between 30 and 60 MPa, which aligns with typical strength classes, although the presence of both low-strength and high-strength concrete in the dataset ensures its applicability across a wide range of concrete applications.

### 3.3. Bias Analysis of the Dataset

The dataset employed in this study is inherently heterogeneous, as it was compiled from diverse experimental programs carried out in different laboratories. Variations in raw material sources, mix design procedures, curing conditions, and testing standards inevitably introduce inconsistencies into the dataset. More importantly, such heterogeneity can result in representation bias, meaning that certain regions of the feature space are either severely underrepresented or entirely missing. This is a critical issue because machine learning models trained on biased datasets may achieve high accuracy on the training samples but demonstrate poor generalization when applied to unseen data. To ensure that the predictive models for RAC elastic modulus are both reliable and generalizable, it is therefore necessary to quantitatively evaluate the degree of bias within the dataset.

**Figure 5:**
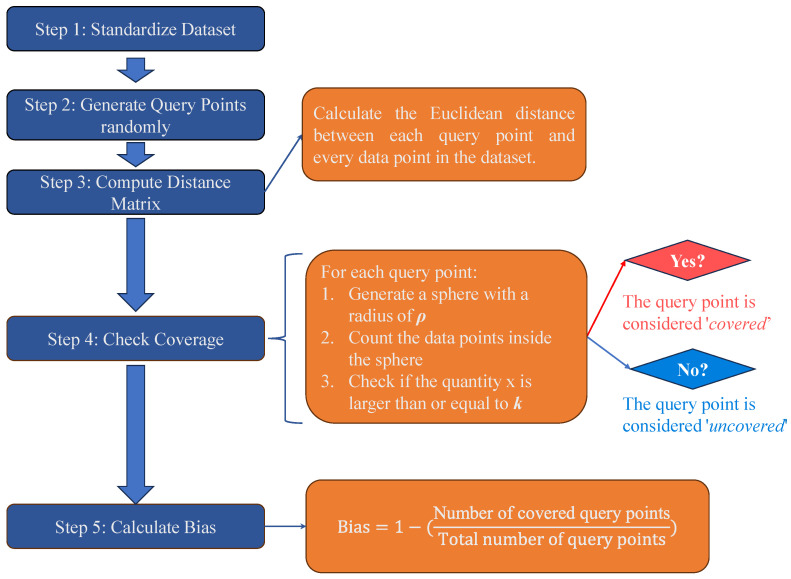
Flowchart of Monte Carlo Bias Estimation (MCBE).

Monte Carlo Bias Estimation (MCBE) was adopted for this purpose [138]. The MCBE algorithm is specifically designed to quantify representation bias, which has become a core challenge for the reliability and generalizability of machine learning models. The fundamental idea of MCBE lies in assessing the degree of dataset coverage within the input feature space, and its specific workflow is illustrated in the flowchart shown in Figure 5. Concretely, the process begins by normalizing all features into the [0, 1] range to ensure comparability across dimensions. A large number of random query points are then generated within this normalized feature space, representing potential unseen data. For each query point, Euclidean distances to all dataset samples are computed, and two parameters—the vicinity radius ρ and the coverage threshold *k*—are used to determine whether the point is covered. A query point is considered covered if at least *k* dataset samples fall within its ρ-neighborhood. The proportion of uncovered query points is then defined as the dataset bias value, ranging between 0 and 1. A larger bias value indicates more severe representation bias and weaker coverage of the feature space. For interpretation, a bias value below 0.2 denotes low bias, values between 0.2 and 0.4 denote moderate bias, values between 0.4 and 0.6 indicate high bias, and values above 0.6 correspond to very high bias. By varying the parameters ρ and *k*, sensitivity analyses can also be performed to obtain a more comprehensive assessment of dataset coverage.

However, it should be noted that the bias analysis can only be conducted with three input features, as high-dimensional data bias analysis is affected by the curse of dimensionality. The curse of dimensionality refers to the phenomenon where, as the number of features increases, the sparsity of the data space dramatically increases, making it difficult for the model to effectively capture the inherent patterns in the data. This not only increases computational complexity but may also lead to inaccurate bias analysis results. Therefore, in predicting the elastic modulus of RAC, we selected three input features from the four available ones: apparent density, water absorption, replacement rate of RA, and compressive strength (*f*_c_), excluding apparent density. The main reason for excluding apparent density is its weak correlation with the elastic modulus across multiple experiments, and its data distribution is inconsistent across different sources of literature, making it less representative in the analysis. Furthermore, the variability of apparent density is high, especially between different data sources, which results in poor stability during model training and introduces noise. It is important to emphasize that the exclusion of apparent density is only for the bias analysis phase, and in other chapters, we still use all four input features for model training and prediction.

**Figure 6:**
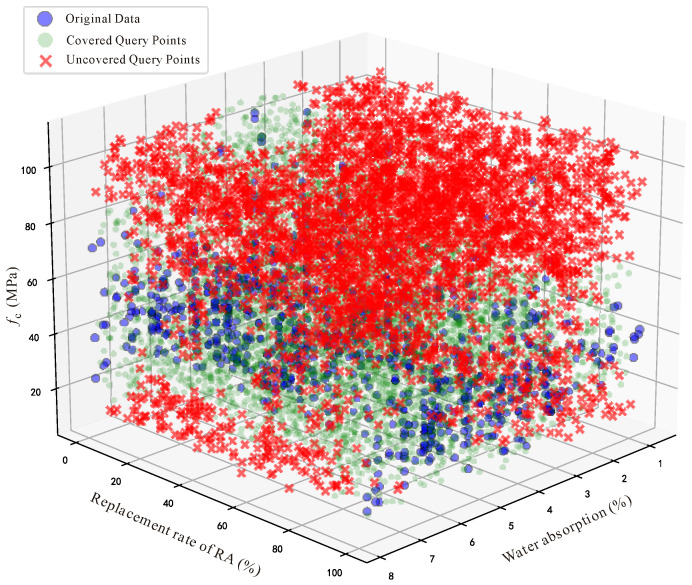
Data coverage visualization in three-dimensional space.

In this study, MCBE was implemented in Python 3.11, directly corresponding to its theoretical formulation. Three features—water absorption, the replacement rate and *f*_c_—are selected for bias analysis, as shown in Figure 6. After normalization, random query points were generated within the three-dimensional space, and distance matrices between query points and dataset samples were calculated. The number of neighboring samples within the radius ρ was counted for each query point, and coverage was determined based on the threshold parameter *k*. The final bias value thus provided a quantitative measure of the dataset’s representativeness in the design space. The advantage of MCBE lies in its statistical foundation: by employing Monte Carlo sampling, it avoids the computational inefficiency of traditional approaches such as Voronoi diagrams, while maintaining flexibility and scalability across datasets of different dimensions.

To evaluate the reliability and generalizability of the model across various datasets, it is essential to perform sensitivity analysis. This step is particularly crucial in understanding how different parameter settings influence the bias values within the dataset. By systematically varying key parameters and analyzing their effects, we can assess the robustness of the model and ensure that the results are not overly sensitive to specific configurations. Table 1 presents the sensitivity analysis of bias values under different parameter settings. The analysis focuses on the influence of two key parameters: vicinity radius

(*ρ*) and coverage threshold (*k*). It is evident that the vicinity radius has a more significant effect on the bias value compared to the coverage threshold. As the vicinity radius decreases, the bias value increases from 0.48 to 0.50, indicating that smaller radii lead to more localized coverage and, potentially, more regions of the feature space being underrepresented. On the other hand, changes in the coverage threshold (*k*) lead to minor fluctuations in bias, especially when the vicinity radius is small.

### 3.4. Bias Removal and Data Preprocessing

After identifying the representation bias in the dataset, it is necessary to implement strategies to mitigate this bias and ensure that the dataset more accurately represents the feature space. Traditional methods, such as Latin Hypercube Sampling (LHS), aim to address this issue by constructing a new, unbiased dataset. However, this approach has significant drawbacks. It discards the original dataset and creates a new one, leading to data wastage. Additionally, to maintain the authenticity of the newly created dataset, it would require extensive re-experiments, which are time-consuming and resource-intensive, especially for large datasets.

In light of these issues, we propose an improvement to the MCBE algorithm, which not only calculates the bias value but also identifies which specific regions of the feature space contribute to this bias. This enhancement allows researchers to focus on collecting data or conducting supplementary experiments in these underrepresented areas rather than completely disregarding the existing dataset. The ability to pinpoint the regions responsible for bias provides a more targeted and efficient solution to the problem of representation bias.

**Figure 7:**
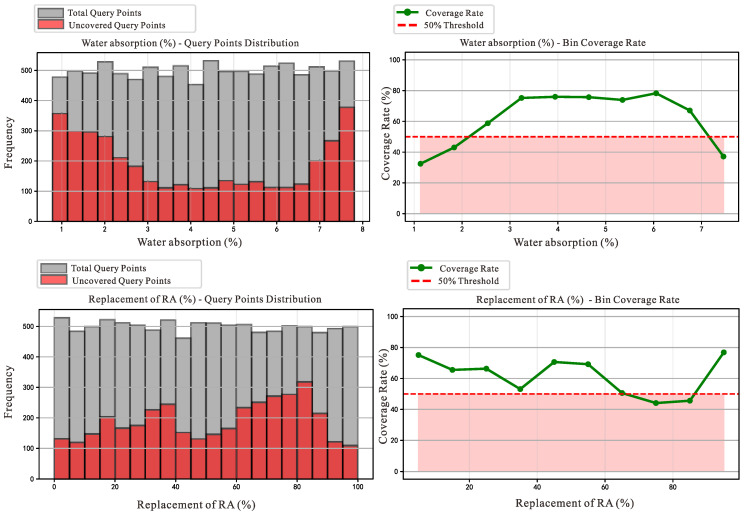

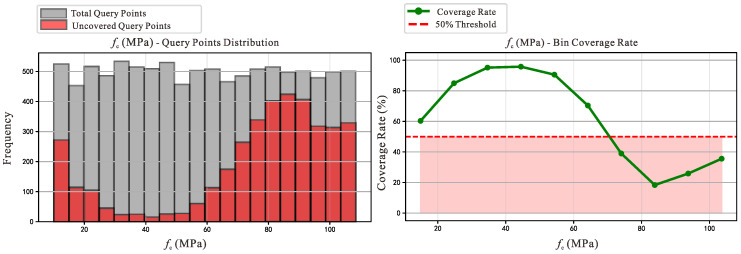
Data before bias removal.

We applied the modified MCBE algorithm to the original dataset, and the results revealed specific intervals within the features of water absorption, replacement rate, and *f*_c_ where data coverage was particularly low, as shown in Table 2, Table 3 and Table 4. To provide a more intuitive understanding of the intervals with low coverage, we created visual representations of the coverage calculated for different intervals. As shown in Figure 7, we set a 50% coverage threshold, and our calculations revealed that the coverage was below this threshold when RAC exhibited high compressive strength. In other words, the RAC data with high compressive strength is the primary cause of the larger bias value in the dataset.

Based on the preceding analysis, the bias in the dataset was predominantly concentrated in the high-strength region, particularly when the compressive strength (*f*_c_) exceeded 78 MPa, where the coverage rate dropped sharply below the 50% threshold, reaching as low as 18.3%. In response to this finding, a targeted data supplementation strategy was implemented: a systematic literature search was conducted on recently published academic papers concerning high-strength RAC, with particular emphasis on experimental data featuring high compressive strengths. Through literature screening, valid samples from 5 high-quality papers were incorporated into the dataset [21,44,139,140,141]. These samples not only covered the high-strength interval but also maintained reasonable distributions across other feature dimensions such as replacement rate and water absorption.

**Figure 8:**
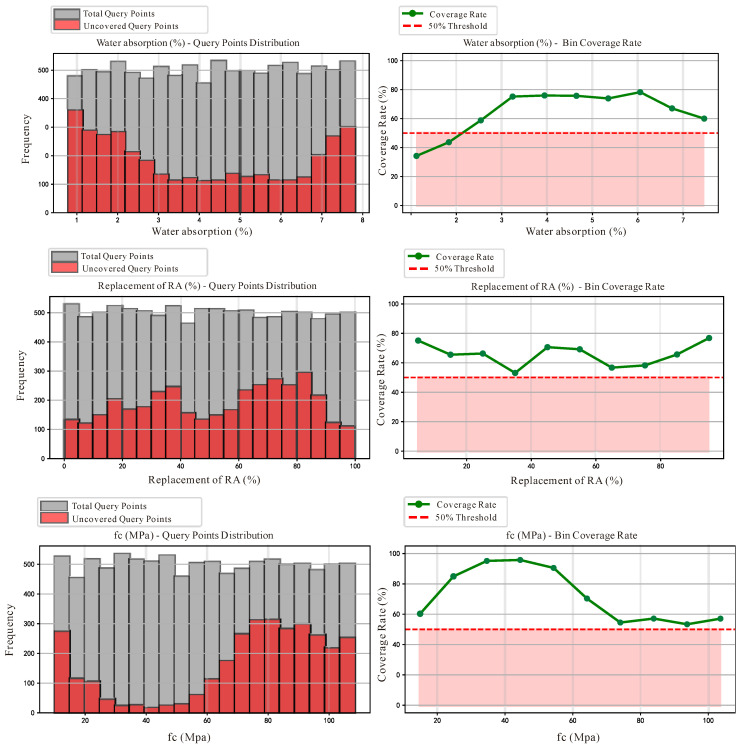
Data after bias removal.

The MCBE analysis was re-performed on the augmented dataset, with results presented in Figure 8. A comparison of the coverage distributions before and after bias removal clearly reveals the following significant improvements:

In the compressive strength (*f*_c_) dimension, the coverage rates in the 78.96–108.51 MPa high-strength interval, which had consistently remained below the 50% threshold, achieved substantial improvement. Regarding the replacement rate distribution, the supplementation of high-strength data generated positive spillover effects, with the coverage rates in the 60–80% intermediate replacement rate intervals also improving and successfully exceeding the 50% threshold. The coverage rate distribution in the water absorption dimension remained relatively stable, indicating that the newly added data maintained favorable feature consistency with the original dataset.

Overall, following the targeted data supplementation and bias removal procedures, the dataset’s bias value underwent a substantial reduction, providing a more balanced and reliable data foundation for subsequent training of machine learning models such as TabPFN. This enhancement particularly strengthens the model’s predictive capability and generalization performance in the domain of high-performance concrete.

## 4. Results and Discussion

Following the completion of outlier analysis and bias removal procedures, and after introducing new data to reduce dataset bias, the augmented dataset was employed to train and evaluate six machine learning models for predicting the elastic modulus of RAC. The dataset was randomly partitioned into training (80%) and testing (20%) subsets to assess the generalization performance of the models. This section presents a comprehensive comparative analysis of the predictive performance across all models, with particular emphasis on TabPFN’s capability to handle heterogeneous small-scale datasets.

The performance of each model was evaluated using three standard statistical metrics. The coefficient of determination (*R*^2^) quantifies the proportion of variance in the dependent variable explained by the model, ranging from 0 to 1, where values closer to 1 indicate superior model fit. It is calculated as:

(13) $$R^{2}=1-\frac{\sum_{i=1}^{n}{\left(y_{i}-{\hat{y}}_{i}\right)}^{2}}{\sum_{i=1}^{n}{\left(y_{i}-\bar{y}\right)}^{2}}$$

where $y_{i}$ denotes the actual value, ${\hat{y}}_{i}$ represents the predicted value, $\bar{y}$ is the mean of actual values, and *n* is the number of samples.

Root mean square error (RMSE) measures the average magnitude of prediction errors, exhibiting greater sensitivity to larger deviations, with units consistent with the original data. It is computed as:

(14) $$RMSE=\;\sqrt{\frac{1}{n}\sum_{i=1}^{n}{\left(y_{i}-{\hat{y}}_{i}\right)}^{2}}$$

Mean absolute error (MAE) represents the arithmetic mean of absolute prediction errors, demonstrating reduced sensitivity to outliers compared to RMSE and providing a more intuitive measure of average prediction deviation. It is expressed as:

(15) $$MAE=\;\frac{1}{n}\sum_{i=1}^{n}\left|y_{i}-{\hat{y}}_{i}\right|$$

**Figure 9:**
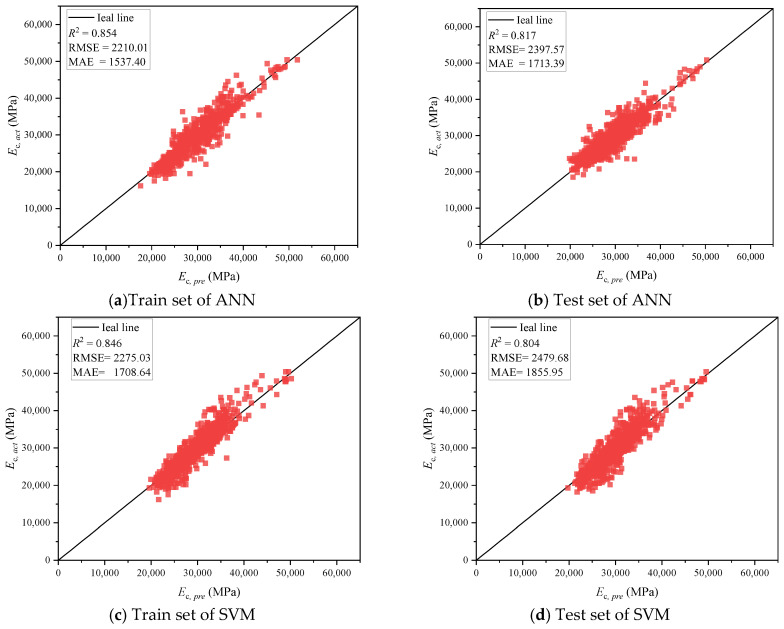

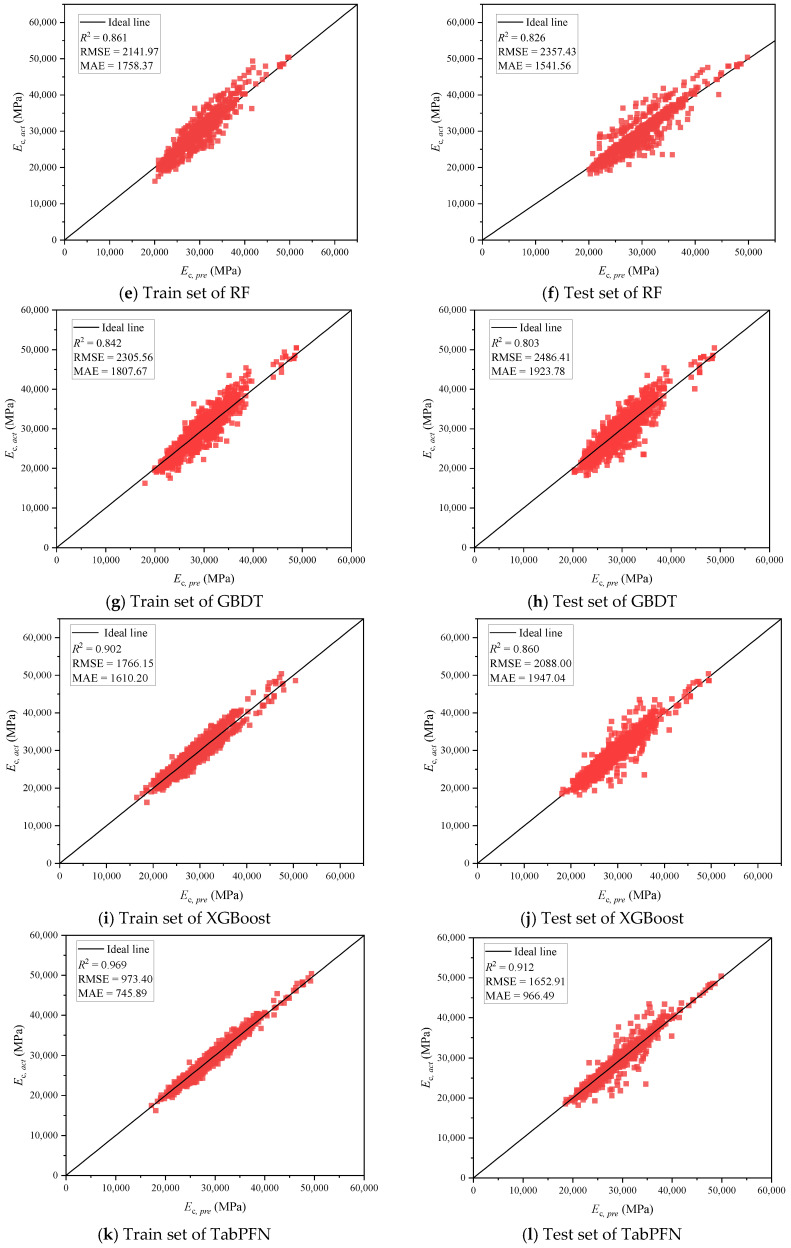
Prediction result from different models.

Figure 9 presents scatter plots comparing predicted versus actual elastic modulus values for all six models across both training and testing datasets. From the overall distribution patterns, traditional machine learning models such as ANN and SVM exhibit considerable dispersion in their predictions, with a substantial proportion of sample points deviating significantly from the ideal line; in contrast, advanced methods such as TabPFN and XGBoost demonstrate tighter fits, with prediction points more concentrated around the ideal line.

Traditional machine learning models (ANN and SVM) exhibit relatively conservative performance. As observed in Figure 9a,b, ANN achieves an *R*^2^ of 0.854 on the training set, which decreases to 0.817 on the test set, suggesting a certain degree of overfitting tendency. The scatter plots reveal that ANN’s predictions in the high elastic modulus region show significant dispersion, with some sample points deviating considerably from the ideal line. This phenomenon may be attributed to the neural network’s difficulty in adequately learning the nonlinear features in this region due to the limited number of high-strength concrete samples. SVM demonstrates similar performance to ANN, as shown in Figure 9c,d, with a training set *R*^2^ of 0.846 and a test set *R*^2^ of 0.804, while RMSE increases to 2479.68 MPa on the test set. Notably, SVM exhibits relatively stable predictions in the low-to-medium elastic modulus region, but displays larger prediction errors in extreme value regions, reflecting the limitations of traditional kernel methods when handling unevenly distributed data.

Ensemble learning methods demonstrate superior predictive performance. Random Forest (RF), as a classical bagging approach, reduces variance by constructing multiple decision trees and averaging their predictions. As shown in Figure 9e,f, RF achieves *R*^2^ = 0.861 on the training set and *R*^2^ = 0.826 on the test set. Although the overall performance surpasses single models, the performance gap between training and test sets indicates that RF still exhibits some overfitting risk. The scatter plots reveal a phenomenon worthy of in-depth investigation: RF achieves high prediction accuracy in the data-dense medium elastic modulus region, with prediction points tightly clustered around the ideal line, while exhibiting larger prediction deviations in data-sparse regions. This regional performance variation not only aligns with RF’s algorithmic characteristic of relying on local training sample density, but more importantly, it directly reflects the persistent influence of the data bias issues identified and addressed in Section 3. Although we have supplemented the severely biased regions with new experimental data, RF’s prediction performance indicates that the augmented dataset still lacks sufficient representativeness in certain regions, and residual bias effects continue to challenge the model’s learning and generalization capabilities. This observation suggests that complete elimination of data bias is a gradual process requiring continuous attention to data accumulation in underrepresented regions. In contrast, TabPFN demonstrates stronger robustness against such residual bias through its pre-trained prior knowledge and Bayesian inference mechanism, which constitutes one of its core advantages on small-scale heterogeneous datasets.

Gradient boosting methods (GBDT and XGBoost) demonstrate stronger learning capabilities through sequentially constructing weak learners and progressively correcting residuals. GBDT shows excellent performance in Figure 9g,h, with training set *R*^2^ = 0.842 and test set *R*^2^ = 0.803, representing the best results among traditional methods. More importantly, GBDT’s prediction points are more uniformly distributed, maintaining good prediction accuracy even in the high elastic modulus region, benefiting from gradient boosting’s progressive residual optimization strategy. XGBoost, as an improved version of GBDT, further enhances performance as shown in Figure 9i,j, achieving a training set *R*^2^ of 0.902 and MAE of only 1610.2 MPa. However, while the test set performance (*R*^2^ = 0.86, RMSE = 1947.04 MPa) is excellent, it still shows some decline compared to the training set. This training-test performance gap suggests that the model complexity may be slightly high, with potential overfitting risk in certain local regions.

TabPFN’s performance is particularly noteworthy, as shown in Figure 9k,l. On the training set, TabPFN achieves an exceptionally high fit with *R*^2^ = 0.969, RMSE of only 973.40 MPa, and MAE of 745.89 MPa, with all metrics significantly superior to other models. More importantly, TabPFN maintains strong performance on the test set with *R*^2^ = 0.912, RMSE of 1652.91 MPa, and MAE of 966.49 MPa. Careful examination of the scatter plots reveals several key features: first, TabPFN’s prediction points are tightly distributed around the ideal line across the entire range, without the extreme region deviation phenomena common in other models; second, the degree of dispersion in prediction points remains consistent across different elastic modulus intervals, indicating that the model possesses stable predictive capability in both data-sparse and data-dense regions; third, the prediction patterns for training and test sets are highly similar, demonstrating that TabPFN has excellent generalization ability without overfitting to specific patterns in the training data.

A common trend can be observed from the comparison of all models: in the low elastic modulus region, all models tend to produce slight overestimation, which may be related to the relatively fewer samples in this region within the dataset and greater variability in quality parameters; in the medium elastic modulus region, which is the most data-dense area, all models demonstrate good prediction accuracy; while in the high elastic modulus region, model performance diverges, with TabPFN and XGBoost maintaining high accuracy, whereas traditional methods show significantly increased prediction deviations. This regional performance variation directly reflects the data bias issues identified in Section 3: despite introducing new data to reduce bias, the representativeness of high-strength concrete samples remains relatively insufficient, posing challenges for model learning.

It is particularly noteworthy that TabPFN’s advantages over XGBoost are reflected not only in the stability of test set performance, but also in achieving excellent performance without requiring complex hyperparameter tuning. Although XGBoost achieves extremely high fit on the training set, this comes at the cost of fine parameter adjustment, and the training-test performance gap suggests potential overfitting risk. In contrast, TabPFN naturally balances fitting capability and generalization performance through its pre-trained transformer architecture and Bayesian inference mechanism, which has important practical significance for application scenarios like RAC where data acquisition costs are high and sample sizes are limited.

Additionally, all models exhibit a degree of heteroscedasticity in the distribution of prediction errors: when elastic modulus is low, prediction errors are relatively small and concentrated; as elastic modulus increases, both the absolute value and dispersion of prediction errors increase. This phenomenon manifests in the figures as greater vertical dispersion of scatter points in high-value regions compared to low-value regions. This may stem from two factors: first, the performance of high-strength RAC is influenced by more microscopic factors, increasing the complexity of material behavior; second, measurement errors for high elastic modulus samples may be relatively larger, introducing additional noise for model learning.

## 5. Shapley Additive Explanations

After evaluating the predictive performance of TabPFN, it is particularly important to conduct an interpretability analysis using methods like SHAP (Shapley Additive Explanations). This is because, although TabPFN has demonstrated excellent accuracy in several predictive tasks, understanding the internal reasoning process of the model is equally crucial. SHAP can reveal the specific contributions of each feature in the model’s prediction, helping us better understand which features have a decisive impact on the prediction results. It also provides assurance for the model’s reliability and interpretability, ensuring its robustness and fairness in practical applications.

SHAP is an interpretability method based on game theory, and its core idea is to calculate the Shapley value of each feature to fairly measure its contribution to the final prediction result. Unlike traditional feature importance methods, SHAP takes into account the interactions and dependencies between features, allowing for a more accurate evaluation of the actual impact of features on the prediction result. Through the calculation of Shapley values, SHAP provides a systematic way to understand the decision-making mechanism of complex machine learning models, especially when dealing with high-dimensional data. Additionally, SHAP provides various visualization tools, such as SHAP dependence plots and partial dependence plots (PDP), which help us analyze the specific impact of features on the prediction result under different feature value changes. This is crucial for further optimizing the model and enhancing its interpretability.

**Figure 10:**
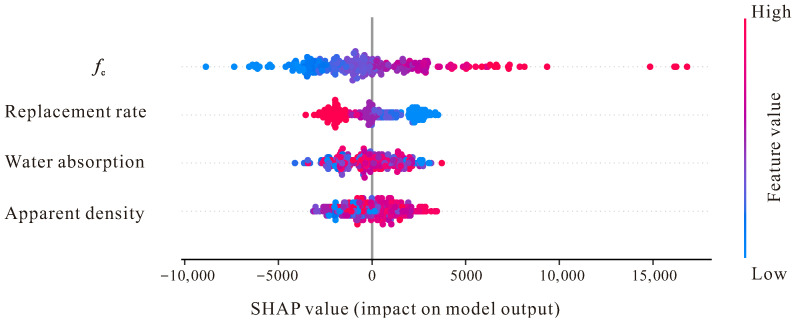
SHAP summary of feature importance.

Based on the analysis of the SHAP Summary of Feature Importance figure, we can observe the overall contribution patterns of various input features to the prediction of recycled aggregate concrete elastic modulus. From Figure 10, it can be seen that compressive strength (*f*_c_) displays the most significant influence, which is highly consistent with the fundamental principles of material mechanics, because there exists a widely recognized statistical correlation between elastic modulus and compressive strength, just as the numerous studies mentioned earlier in the paper have established a significant correlation between *E*_c_ and *f*_c_. Each point in the figure represents a sample, and the color encoding reflects the magnitude of feature values. Through observation, it can be found that high compressive strength values (red points) are obviously concentrated in the positive SHAP value region, which indicates that higher compressive strength will significantly increase the predicted value of elastic modulus, while low compressive strength values (blue points) are mainly distributed in the negative SHAP value region, leading to a decrease in the predicted elastic modulus.

The influence of replacement rate is also relatively prominent, and its SHAP value distribution presents obvious bidirectional characteristics, which reflects the bimodal distribution characteristics of 0% and 100% replacement rates existing in the paper’s dataset. When the replacement rate is high, it usually leads to a decrease in elastic modulus due to the presence of old mortar layer, increase in microcracks, and decline in interfacial transition zone quality, which is manifested in the SHAP figure as high replacement rate corresponding to negative SHAP contribution values. The influence pattern of water absorption likewise conforms to expectations; higher water absorption usually indicates poorer recycled aggregate quality and higher porosity, thereby weakening the overall stiffness of concrete, therefore high-water absorption values mainly correspond to negative SHAP values. Although apparent density also participates in the prediction, its influence is relatively small and scattered, which echoes the decision in Section 3.3 of the paper to exclude this feature during the bias analysis stage, indicating that the correlation between apparent density and elastic modulus does not perform stably in the dataset.

**Figure 11:**
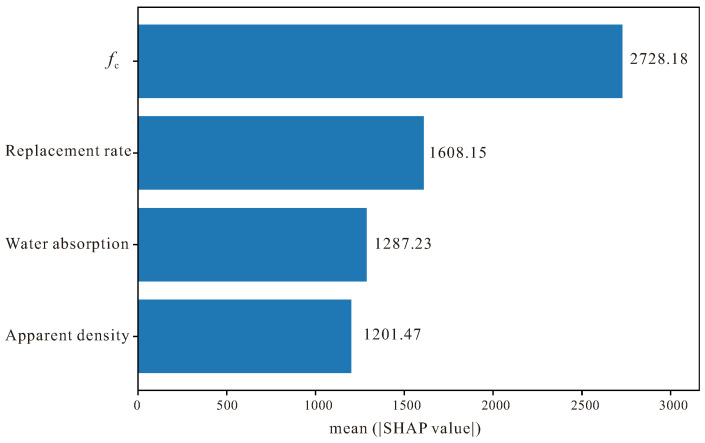
Mean SHAP values.

Through calculating the mean absolute SHAP value of each feature, Figure 11 provides a more intuitive and quantified feature importance ranking perspective. This figure clearly displays the average contribution magnitude of each input feature to elastic modulus prediction, and its ranking results further confirm that compressive strength occupies an absolute dominant position among all features, with its mean SHAP value significantly higher than other features, which once again confirms the core position of *f*_c_ as the most critical indicator for predicting *E*_c_. Compared with the Summary figure, the Mean SHAP figure, through taking absolute values and calculating averages, eliminates the differences in positive and negative directions, and measures feature importance purely from the perspective of contribution magnitude, therefore it can more clearly identify which features have the most significant impact on model decisions.

From the figure, it can be seen that replacement rate ranks second, which indicates that although its influence direction may vary with specific values, its overall perturbation degree to prediction results is quite considerable, which is closely related to RAC material characteristics, because replacement rate directly determines the proportion of recycled aggregate in concrete and thus affects the microstructure and macroscopic performance of the material. Water absorption ranks third, and although its average contribution value is lower than replacement rate, it still has a non-negligible influence, which reflects the important role of recycled aggregate quality on elastic modulus, because water absorption is one of the key indicators for evaluating RA quality grade. The mean SHAP value of apparent density is the smallest, which further verifies the relatively secondary position of this feature in this dataset, and this is consistent with the observation results mentioned earlier that the apparent density data distribution is inconsistent and stability is poor.

**Figure 12:**
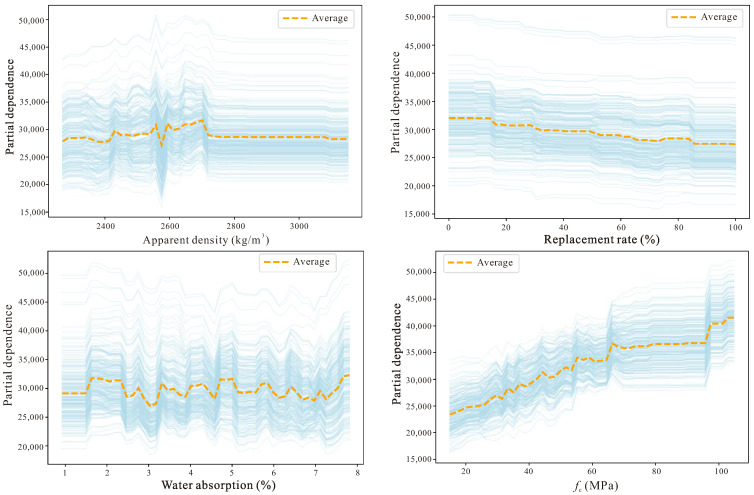
SHAP interaction effects (ICE Plots).

Figure 12 reveals the complex interaction effects between features and the heterogeneous response patterns of individual samples to feature changes, which is crucial for deeply understanding how the TabPFN model captures the nonlinear relationships of RAC material performance. Different from the previous two figures, the ICE plot not only displays the overall importance of features, but further reveals how the predicted values of different samples present differentiated response trajectories when a certain feature changes, and this sample-level fine-grained analysis can help us identify which feature combinations will produce synergistic or antagonistic effects. From the figure it can be observed that each thin line represents an independent sample, and when moving along a certain feature axis, the direction and slope changes of these lines reflect the marginal impact of this feature on the prediction results of different samples, while the degree of divergence or convergence between lines indicates the intensity of feature interaction.

Particularly worthy of attention is the possible interaction effect between compressive strength and replacement rate, because for concrete of different strength grades, the influence of replacement rate may exhibit different sensitivities, high-strength concrete may have more stringent requirements for recycled aggregate quality, therefore the performance decline is more significant at high replacement rates, and this nonlinear interaction relationship will be manifested in the ICE plot as lines with different starting points presenting different decline rates when replacement rate increases. Similarly, the interaction between water absorption and replacement rate also has important significance, because recycled aggregate with high water absorption will produce a superimposed negative impact on elastic modulus under high replacement rate conditions, and this effect is not a simple linear superposition, but is realized through the comprehensive degradation of interfacial transition zone quality. In addition, the ICE plot can also identify abnormal samples or special behavior patterns in the dataset, for example, the response curves of some samples obviously deviate from mainstream trends, which may suggest that specific material mix proportions or test conditions have produced unique performance manifestations.

**Figure 13:**
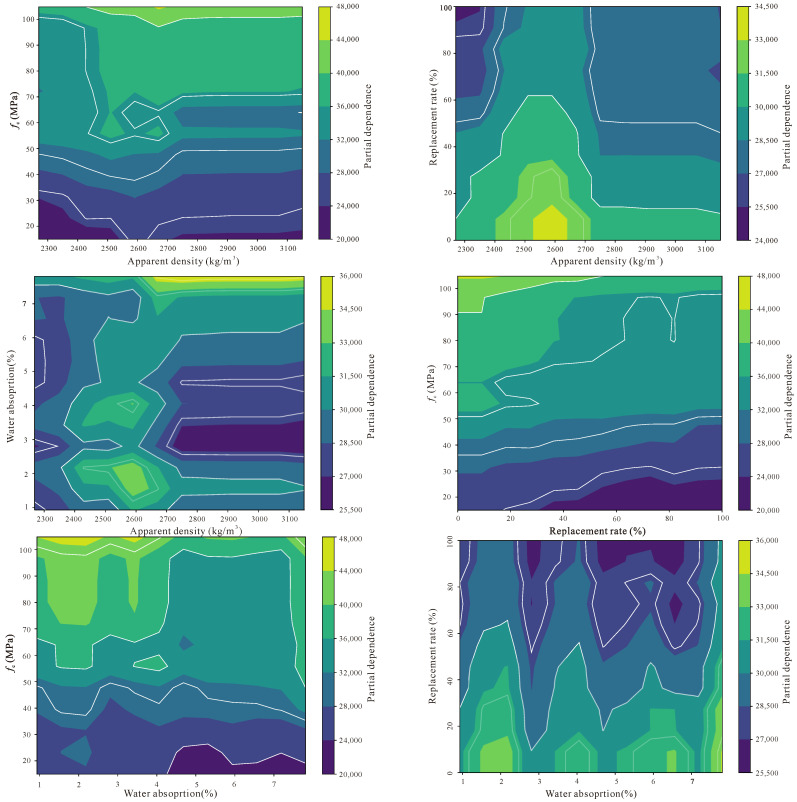
Partial dependence plots.

The Partial Dependence Plots (PDP) figure (Figure 13), through marginalizing all samples, displays the average influence trend of a single feature on elastic modulus predicted values when it changes within its value range, and this global perspective analysis method can effectively strip away the interference of other features, enabling us to clearly see the pure relationship between each feature and the target variable. Different from the ICE plot focusing on the heterogeneous responses of individual samples, PDP, through averaging the predicted values of all samples, provides a population-level feature effect description, therefore it is more suitable for identifying the dominant trends of feature influence and potential nonlinear patterns. From the figure it can be observed that the PDP curve of compressive strength should present an obvious positive upward trend, which is completely consistent with the fundamental laws of material mechanics, that is, as the compressive strength of concrete increases, its ability to resist deformation also correspondingly strengthens, thereby leading to an increase in elastic modulus, and moreover this relationship is very likely not a simple linear relationship, and may exhibit different growth rates in the low-strength and high-strength intervals, reflecting the differential evolution laws of concrete internal microstructure at different strength grades.

The PDP curve of replacement rate is expected to show an overall trend of elastic modulus decrease as the recycled aggregate replacement rate increases, but the shape of this curve may not be monotonically decreasing, but rather exhibits relatively gentle changes in certain replacement rate intervals, which may be related to the bimodal distribution characteristics of high proportions of 0% and 100% replacement rate samples in the dataset, and may also suggest the existence of a certain critical replacement rate, before which the performance decline is relatively mild, and after exceeding this threshold the decline speed obviously accelerates. The PDP curve of water absorption should present a negative influence because higher water absorption directly indicates the development degree of the internal pore structure of recycled aggregate and the quality degradation of adhered old mortar, and these factors will all weaken the compactness and continuity of the concrete matrix and thus reduce its overall stiffness. From the slope changes of the curve, it can also be determined whether the sensitivity of water absorption to elastic modulus at different numerical intervals has differences; for example, when water absorption changes from low to medium levels, the influence may be relatively gentle, while when water absorption enters the high value zone the influence may be sharply amplified.

Although the PDP curve of apparent density should theoretically show a positive correlation relationship, because higher density usually means more compact material structure, the actual curve may exhibit a weaker and insufficiently stable trend, which once again confirms the limitations of this feature in the current dataset, possibly because the differences in testing methods from different literature sources or the complex composition of recycled aggregate itself has led to the blurring of the relationship between apparent density and elastic modulus.

The SHAP analysis clearly identifies compressive strength (*f*_c_) as the dominant predictor of the elastic modulus (*E*_c_) of RAC, aligning closely with the fundamental mechanics of composite materials. This strong correspondence between the data-driven and physical domains underscores the interpretability and reliability of the TabPFN model. The replacement ratio (*RR*) exhibits a bidirectional influence, reflecting the bimodal distribution of the dataset—moderate replacement enhances stiffness through improved packing, whereas excessive replacement deteriorates *E*_c_ due to the weaker recycled aggregate skeleton. Water absorption (WA), a direct indicator of aggregate quality, exerts a consistently negative contribution, while apparent density shows limited predictive strength, likely due to inconsistencies in measurement and reporting across data sources.

The ICE plots reveal marked heterogeneity in sample responses and emphasize the presence of nonlinear interactions among variables. Meanwhile, the PDP curves further confirm a nonlinear positive relationship between *f*_c_ and *E*_c_, as well as the differentiated influence patterns of RR and WA across their respective ranges.

Collectively, these interpretability analyses not only validate the physical soundness of the TabPFN predictions but also provide actionable insights for RAC mix design and quality control—highlighting the need to optimize aggregate replacement and water absorption parameters to achieve balanced stiffness and sustainability.

## 6. Conclusions

This study establishes a robust and transferable predictive framework for estimating the elastic modulus (*E*_c_) of RAC, integrating the Tabular Prior-data Fitted Network (TabPFN) with Monte Carlo Bias Estimation (MCBE). The approach addresses the two long-standing bottlenecks in data-driven materials modeling—dataset bias and limited generalization—by combining a foundation-model learning paradigm with probabilistic bias quantification. A database containing 1161 records from diverse experimental sources was curated to enable systematic bias analysis and cross-domain model validation. From this study, the following conclusions can be drawn:

(1) This work represents the first application of a transformer-based foundation model to RAC. Leveraging pre-training on millions of Bayesians and causal inference tasks, TabPFN performs one-shot Bayesian prediction on small and heterogeneous tabular datasets. It eliminates the need for task-specific retraining or hyperparameter tuning, enabling robust inference even under data scarcity. This establishes a transferable learning framework that can generalize across varying experimental and regional data distributions;
(2) MCBE analysis revealed substantial representational gaps—particularly within the high-strength RAC domain—where traditional models suffered accuracy drops exceeding 25%. Through targeted data augmentation guided by MCBE metrics, these biases were significantly mitigated, leading to uniform performance across all strength ranges. The synergy between TabPFN’s prior-informed generalization and MCBE’s bias quantification yielded a bias-aware predictive model capable of maintaining accuracy in previously underrepresented data domains.
(3) Compared with five benchmark machine learning algorithms (ANN, SVM, RF, GBDT, and XGBoost), TabPFN achieved the highest predictive performance (*R*^2^ = 0.912, RMSE = 1.65 GPa) with minimal computational cost. Predictions were generated in a single forward pass, reducing training time by several orders of magnitude. The model’s stability across heterogeneous datasets confirms its robustness and transferability, enabling consistent results across diverse experimental settings without dataset-specific calibration.
(4) SHAP-based analysis verified that compressive strength dominates *E*_c_ prediction, followed by the influence of aggregate quality indicators such as replacement ratio and water absorption. These patterns are mechanically consistent with established theories of stiffness degradation in recycled aggregate systems, confirming that the TabPFN framework maintains not only predictive accuracy but also physical interpretability.

Overall, the combined TabPFN–MCBE framework provides a scalable pathway for developing reliable and transferable AI tools in construction materials science. It offers a general methodology for quantifying and correcting data bias, while enabling uncertainty-aware prediction and rapid deployment under limited-data conditions. Beyond predicting *E*_c_, the proposed paradigm can be extended to other performance indicators—such as tensile strength, shrinkage, and chloride penetration—supporting the transition toward data-driven, risk-informed, and performance-based design of sustainable concrete structures.

## Figures and Tables

**Table 1:** Sensitivity analysis of bias values under different parameter settings.

ρ	*k*	Bias
0.2	3	0.510
0.1	3	0.483
0.05	3	0.512
0.01	3	0.479
0.01	5	0.495

**Table 2:** Water absorption.

Interval Range	Total Number of Query Points	Number of Covered Points	Coverage Rate (%)
[0.78, 1.48]	977	317	32.4
[1.48, 2.19]	1020	439	43.0
[2.19, 2.89]	961	565	58.8
[2.89, 3.59]	993	747	75.2
[3.59, 4.29]	970	737	76.0
[4.29, 5.00]	1026	777	75.7
[5.00, 5.70]	986	729	73.9
[5.70, 6.40]	1040	814	78.3
[6.40, 7.11]	1002	672	67.1
[7.11, 7.81]	1025	381	37.2

**Table 3:** Replacement rate of RA.

Interval Range	Total Number of Query Points	Number of Covered Points	Coverage Rate (%)
[0, 10]	1013	761	75.1
[10, 20]	1021	669	65.5
[20, 30]	1018	675	66.3
[30, 40]	1011	537	53.1
[40, 50]	974	688	70.6
[50, 60]	1016	703	69.2
[60, 70]	988	500	50.6
[70, 80]	986	435	44.1
[80, 90]	980	447	45.6
[90, 100]	993	763	76.8

**Table 4:** Compressive strength.

Interval Range	Total Number of Query Points	Number of Covered Points	Coverage Rate (%)
[10.00, 19.85]	980	389	60.3
[19.85, 29.70]	1003	151	84.9
[29.70, 39.55]	1051	51	95.1
[39.55, 49.40]	1039	44	95.8
[49.40, 59.26]	962	91	90.5
[59.26, 69.11]	975	289	70.4
[69.11, 78.96]	993	606	39.0
[78.96, 88.81]	1014	828	18.3
[88.81, 98.66]	982	728	25.9
[98.66, 108.51]	1001	645	35.6

## Data Availability

The original contributions presented in this study are included in the article. Further inquiries can be directed to the corresponding author.

## References

[1] Trivedi S.S., Snehal K., Das B., Barbhuiya S. (2023). A Comprehensive Review towards Sustainable Approaches on the Processing and Treatment of Construction and Demolition Waste. Constr. Build. Mater..

[2] Zhang C., Hu M., Di Maio F., Sprecher B., Yang X., Tukker A. (2022). An Overview of the Waste Hierarchy Framework for Analyzing the Circularity in Construction and Demolition Waste Management in Europe. Sci. Total Environ..

[3] Duan H., Li J. (2016). Construction and Demolition Waste Management: China’s Lessons. Waste Manag. Res..

[4] Moschen-Schimek J., Kasper T., Huber-Humer M. (2023). Critical Review of the Recovery Rates of Construction and Demolition Waste in the European Union–An Analysis of Influencing Factors in Selected EU Countries. Waste Manag..

[5] Rayhan D.S.A., Bhuiyan I.U. (2024). Review of Construction and Demolition Waste Management Tools and Frameworks with the Classification, Causes, and Impacts of the Waste. Waste Dispos. Sustain. Energy.

[6] Kabirifar K., Mojtahedi M., Wang C., Tam V.W. (2020). Construction and Demolition Waste Management Contributing Factors Coupled with Reduce, Reuse, and Recycle Strategies for Effective Waste Management: A Review. J. Clean. Prod..

[7] Zhang Y., Luo W., Wang J., Wang Y., Xu Y., Xiao J. (2019). A Review of Life Cycle Assessment of Recycled Aggregate Concrete. Constr. Build. Mater..

[8] Marinković S., Radonjanin V., Malešev M., Ignjatović I. (2010). Comparative Environmental Assessment of Natural and Recycled Aggregate Concrete. Waste Manag..

[9] Wang B., Yan L., Fu Q., Kasal B. (2021). A Comprehensive Review on Recycled Aggregate and Recycled Aggregate Concrete. Resour. Conserv. Recycl..

[10] Silva R., De Brito J., Dhir R.K. (2017). Availability and Processing of Recycled Aggregates within the Construction and Demolition Supply Chain: A Review. J. Clean. Prod..

[11] Vahidi A., Mostaani A., Gebremariam A.T., Di Maio F., Rem P. (2024). Feasibility of Utilizing Recycled Coarse Aggregates in Commercial Concrete Production. J. Clean. Prod..

[12] Xiao J., Zhang K., Ding T., Zhang Q., Xiao X. (2023). Fundamental Issues towards Unified Design Theory of Recycled and Natural Aggregate Concrete Components. Engineering.

[13] Remišová E., Deckỳ M., Mikolaš M., Hájek M., Kovalčík L., Mečár M. (2016). Design of Road Pavement Using Recycled Aggregate. IOP Conf. Ser. Earth Environ. Sci..

[14] Tam V.W., Soomro M., Evangelista A.C.J. (2018). A Review of Recycled Aggregate in Concrete Applications (2000–2017). Constr. Build. Mater..

[15] Bai G., Zhu C., Liu C., Liu B. (2020). An Evaluation of the Recycled Aggregate Characteristics and the Recycled Aggregate Concrete Mechanical Properties. Constr. Build. Mater..

[16] Xiao J., Li W., Fan Y., Huang X. (2012). An Overview of Study on Recycled Aggregate Concrete in China (1996–2011). Constr. Build. Mater..

[17] Liang C., Bao J., Gu F., Lu J., Ma Z., Hou S., Duan Z. (2025). Determining the Importance of Recycled Aggregate Characteristics Affecting the Elastic Modulus of Concrete by Modeled Recycled Aggregate Concrete: Experiment and Numerical Simulation. Cem. Concr. Compos..

[18] Chen X., Hao H., de Brito J., Liu G., Wang J. (2025). Discussion of the Implementation of Water Compensation Methods for Recycled Aggregate Concrete: A Critical Review. Cem. Concr. Compos..

[19] Thomas C., Setién J., Polanco J.A., Alaejos P., De Juan M.S. (2013). Durability of Recycled Aggregate Concrete. Constr. Build. Mater..

[20] Guo H., Shi C., Guan X., Zhu J., Ding Y., Ling T.-C., Zhang H., Wang Y. (2018). Durability of Recycled Aggregate Concrete–A Review. Cem. Concr. Compos..

[21] Ozbakkaloglu T., Gholampour A., Xie T. (2018). Mechanical and Durability Properties of Recycled Aggregate Concrete: Effect of Recycled Aggregate Properties and Content. J. Mater. Civ. Eng..

[22] Lotfi S., Eggimann M., Wagner E., Mróz R., Deja J. (2015). Performance of Recycled Aggregate Concrete Based on a New Concrete Recycling Technology. Constr. Build. Mater..

[23] Qin J., Geng Y., Chang Y.-C., Zhang H., Wang Y.-Y. (2025). Probabilistic Model for Compressive Strength of Recycled Aggregate Concrete Accounting for Uncertainty of Recycled Aggregates from Different Sources. Constr. Build. Mater..

[24] Wang J., Zhang J., Cao D., Dang H., Ding B. (2020). Comparison of Recycled Aggregate Treatment Methods on the Performance for Recycled Concrete. Constr. Build. Mater..

[25] Butler L., West J.S., Tighe S.L. (2013). Effect of Recycled Concrete Coarse Aggregate from Multiple Sources on the Hardened Properties of Concrete with Equivalent Compressive Strength. Constr. Build. Mater..

[26] Kou S., Poon C. (2015). Effect of the Quality of Parent Concrete on the Properties of High Performance Recycled Aggregate Concrete. Constr. Build. Mater..

[27] Xiao J., Poon C.S., Wang Y., Zhao Y., Ding T., Geng Y., Ye T., Li L. (2022). Fundamental Behaviour of Recycled Aggregate Concrete–Overview I: Strength and Deformation. Mag. Concr. Res..

[28] Etxeberria M., Vázquez E., Marí A., Barra M. (2007). Influence of Amount of Recycled Coarse Aggregates and Production Process on Properties of Recycled Aggregate Concrete. Cem. Concr. Res..

[29] Medina C., Zhu W., Howind T., de Rojas M.I.S., Frías M. (2014). Influence of Mixed Recycled Aggregate on the Physical–Mechanical Properties of Recycled Concrete. J. Clean. Prod..

[30] Poon C.S., Shui Z., Lam L., Fok H., Kou S. (2004). Influence of Moisture States of Natural and Recycled Aggregates on the Slump and Compressive Strength of Concrete. Cem. Concr. Res..

[31] Etxeberria M., Marí A.R., Vázquez E. (2007). Recycled Aggregate Concrete as Structural Material. Mater. Struct..

[32] Silva R.V., De Brito J., Dhir R. (2015). Tensile Strength Behaviour of Recycled Aggregate Concrete. Constr. Build. Mater..

[33] Gayarre F.L., Perez C.L.-C., Lopez M.A.S., Cabo A.D. (2014). The Effect of Curing Conditions on the Compressive Strength of Recycled Aggregate Concrete. Constr. Build. Mater..

[34] Tam V.W., Wang K., Tam C.M. (2008). Assessing Relationships among Properties of Demolished Concrete, Recycled Aggregate and Recycled Aggregate Concrete Using Regression Analysis. J. Hazard. Mater..

[35] Xiao J.-Z., Li J.-B., Zhang C. (2006). On Relationships between the Mechanical Properties of Recycled Aggregate Concrete: An Overview. Mater. Struct..

[36] Duan Z.H., Poon C.S. (2014). Properties of Recycled Aggregate Concrete Made with Recycled Aggregates with Different Amounts of Old Adhered Mortars. Mater. Des..

[37] Casuccio M., Torrijos M., Giaccio G., Zerbino R. (2008). Failure Mechanism of Recycled Aggregate Concrete. Constr. Build. Mater..

[38] Hansen T.C. (1992). Recycling of Demolished Concrete and Masonry.

[39] Katz A. (2003). Properties of Concrete Made with Recycled Aggregate from Partially Hydrated Old Concrete. Cem. Concr. Res..

[40] Verian K.P., Ashraf W., Cao Y. (2018). Properties of Recycled Concrete Aggregate and Their Influence in New Concrete Production. Resour. Conserv. Recycl..

[41] Kim J. (2022). Influence of Quality of Recycled Aggregates on the Mechanical Properties of Recycled Aggregate Concretes: An Overview. Constr. Build. Mater..

[42] Wang D., Lu C., Zhu Z., Zhang Z., Liu S., Ji Y., Xing Z. (2023). Mechanical Performance of Recycled Aggregate Concrete in Green Civil Engineering. Case Stud. Constr. Mater..

[43] Tijani A.I., Yang J., Dirar S. (2015). Enhancing the Performance of Recycled Aggregate Concrete with Microsilica. Int. J. Struct. Civ. Eng. Res..

[44] Andreu G., Miren E. (2014). Experimental Analysis of Properties of High Performance Recycled Aggregate Concrete. Constr. Build. Mater..

[45] Bui N.K., Satomi T., Takahashi H. (2017). Improvement of Mechanical Properties of Recycled Aggregate Concrete Basing on a New Combination Method between Recycled Aggregate and Natural Aggregate. Constr. Build. Mater..

[46] Wardeh G., Ghorbel E., Gomart H. (2015). Mix Design and Properties of Recycled Aggregate Concretes: Applicability of Eurocode 2. Int. J. Concr. Struct. Mater..

[47] Hamad B.S., Dawi A.H. (2017). Sustainable Normal and High Strength Recycled Aggregate Concretes Using Crushed Tested Cylinders as Coarse Aggregates. Case Stud. Constr. Mater..

[48] Abreu V., Evangelista L., De Brito J. (2018). The Effect of Multi-Recycling on the Mechanical Performance of Coarse Recycled Aggregates Concrete. Constr. Build. Mater..

[49] Cho S.-K., Kim G.-Y., Eu H.-M., Kim Y.-R., Lee C.-M. (2021). The Effect of Recycled Aggregate Produced by the New Crushing Device with Multi-Turn Wings and Guide Plate on the Mechanical Properties and Carbonation Resistance of Concrete. J. Korean Recycl. Constr. Resour. Inst..

[50] Fonseca N., De Brito J., Evangelista L. (2011). The Influence of Curing Conditions on the Mechanical Performance of Concrete Made with Recycled Concrete Waste. Cem. Concr. Compos..

[51] Zhang H., Wang Y., Lehman D.E., Geng Y., Kuder K. (2020). Time-Dependent Drying Shrinkage Model for Concrete with Coarse and Fine Recycled Aggregate. Cem. Concr. Compos..

[52] Han T., Siddique A., Khayat K., Huang J., Kumar A. (2020). An Ensemble Machine Learning Approach for Prediction and Optimization of Modulus of Elasticity of Recycled Aggregate Concrete. Constr. Build. Mater..

[53] Kazmi S.M.S., Munir M.J., Wu Y.-F., Lin X., Ashiq S.Z. (2023). Development of Unified Elastic Modulus Model of Natural and Recycled Aggregate Concrete for Structural Applications. Case Stud. Constr. Mater..

[54] Behnood A., Olek J., Glinicki M.A. (2015). Predicting Modulus Elasticity of Recycled Aggregate Concrete Using M5′ Model Tree Algorithm. Constr. Build. Mater..

[55] Duan Z.-H., Kou S.-C., Poon C.-S. (2013). Using Artificial Neural Networks for Predicting the Elastic Modulus of Recycled Aggregate Concrete. Constr. Build. Mater..

[56] Ye H.-J., Liu S.-Y., Chao W.-L. (2025). A Closer Look at Tabpfn v2: Strength, Limitation, and Extension. arXiv.

[57] Hollmann N., Müller S., Purucker L., Krishnakumar A., Körfer M., Hoo S.B., Schirrmeister R.T., Hutter F. (2025). Accurate Predictions on Small Data with a Tabular Foundation Model. Nature.

[58] Hollmann N., Müller S., Eggensperger K., Hutter F. (2022). Tabpfn: A Transformer That Solves Small Tabular Classification Problems in a Second. arXiv.

[59] Yu Z., Yu R., Ge X., Fu J., Hu Y., Chen S. (2025). Tabular Prior-Data Fitted Network for Urban Air Temperature Inference and High Temperature Risk Assessment. Sustain. Cities Soc..

[60] Friedman J.H. (2001). Greedy Function Approximation: A Gradient Boosting Machine. Ann. Stat..

[61] Arezoumandi M., Smith A., Volz J.S., Khayat K.H. (2015). An Experimental Study on Flexural Strength of Reinforced Concrete Beams with 100% Recycled Concrete Aggregate. Eng. Struct..

[62] Dilbas H., Şimşek M., Çakır Ö. (2014). An Investigation on Mechanical and Physical Properties of Recycled Aggregate Concrete (RAC) with and without Silica Fume. Constr. Build. Mater..

[63] Eguchi K., Teranishi K., Nakagome A., Kishimoto H., Shinozaki K., Narikawa M. (2007). Application of Recycled Coarse Aggregate by Mixture to Concrete Construction. Constr. Build. Mater..

[64] Pickel D., Tighe S., West J.S. (2017). Assessing Benefits of Pre-Soaked Recycled Concrete Aggregate on Variably Cured Concrete. Constr. Build. Mater..

[65] Xuan D., Zhan B., Poon C.S. (2016). Assessment of Mechanical Properties of Concrete Incorporating Carbonated Recycled Concrete Aggregates. Cem. Concr. Compos..

[66] Assaad J., Daou Y. (2017). Behavior of Structural Polymer-Modified Concrete Containing Recycled Aggregates. J. Adhes. Sci. Technol..

[67] Kim S.-W., Yun H.-D., Park W.-S., Jang Y.-I. (2015). Bond Strength Prediction for Deformed Steel Rebar Embedded in Recycled Coarse Aggregate Concrete. Mater. Des..

[68] Luo S., Ye S., Xiao J., Zheng J., Zhu Y. (2018). Carbonated Recycled Coarse Aggregate and Uniaxial Compressive Stress-Strain Relation of Recycled Aggregate Concrete. Constr. Build. Mater..

[69] Suryawanshi S., Singh B., Bhargava P. (2015). Characterization of Recycled Aggregate Concrete. Advances in Structural Engineering: Materials, Volume Three.

[70] Choi W.-C., Yun H.-D. (2012). Compressive Behavior of Reinforced Concrete Columns with Recycled Aggregate under Uniaxial Loading. Eng. Struct..

[71] Hu X., Lu Q., Xu Z., Zhang W., Cheng S. (2018). Compressive Stress-Strain Relation of Recycled Aggregate Concrete under Cyclic Loading. Constr. Build. Mater..

[72] González B., Martínez F. (2008). Concretes with Aggregates from Demolition Waste and Silica Fume. Mater. Mech. Prop. Build. Environ..

[73] Adams M.P., Fu T., Cabrera A.G., Morales M., Ideker J.H., Isgor O.B. (2016). Cracking Susceptibility of Concrete Made with Coarse Recycled Concrete Aggregates. Constr. Build. Mater..

[74] Knaack A.M., Kurama Y.C. (2015). Creep and Shrinkage of Normal-Strength Concrete with Recycled Concrete Aggregates. ACI Mater. J..

[75] Geng Y., Wang Y., Chen J. (2016). Creep Behaviour of Concrete Using Recycled Coarse Aggregates Obtained from Source Concrete with Different Strengths. Constr. Build. Mater..

[76] Geng Y., Zhao M., Yang H., Wang Y. (2019). Creep Model of Concrete with Recycled Coarse and Fine Aggregates That Accounts for Creep Development Trend Difference between Recycled and Natural Aggregate Concrete. Cem. Concr. Compos..

[77] Li L., Xuan D., Sojobi A.O., Liu S., Chu S., Poon C.S. (2021). Development of Nano-Silica Treatment Methods to Enhance Recycled Aggregate Concrete. Cem. Concr. Compos..

[78] Çakır Ö., Dilbas H. (2021). Durability Properties of Treated Recycled Aggregate Concrete: Effect of Optimized Ball Mill Method. Constr. Build. Mater..

[79] Beltrán M.G., Barbudo A., Agrela F., Galvín A.P., Jiménez J.R. (2014). Effect of Cement Addition on the Properties of Recycled Concretes to Reach Control Concretes Strengths. J. Clean. Prod..

[80] Fan Y., Xiao J., Tam V.W. (2014). Effect of Old Attached Mortar on the Creep of Recycled Aggregate Concrete. Struct. Concr..

[81] Shaban W.M., Elbaz K., Yang J., Thomas B.S., Shen X., Li L., Du Y., Xie J., Li L. (2021). Effect of Pozzolan Slurries on Recycled Aggregate Concrete: Mechanical and Durability Performance. Constr. Build. Mater..

[82] Yang I.-H., Jeong J.-Y. (2016). Effect of Recycled Coarse Aggregate on Compressive Strength and Mechanical Properties of Concrete. J. Korea Concr. Inst..

[83] Gonzalez-Fonteboa B., Martinez-Abella F., Eiras-Lopez J., Seara-Paz S. (2011). Effect of Recycled Coarse Aggregate on Damage of Recycled Concrete. Mater. Struct..

[84] Luo S., Wu W., Wu K. (2018). Effect of Recycled Coarse Aggregates Enhanced by CO_2_ on the Mechanical Properties of Recycled Aggregate Concrete. IOP Conf. Ser. Mater. Sci. Eng..

[85] Kachouh N., El-Hassan H., El-Maaddawy T. (2019). Effect of Steel Fibers on the Performance of Concrete Made with Recycled Concrete Aggregates and Dune Sand. Constr. Build. Mater..

[86] Huang Y., He X., Sun H., Sun Y., Wang Q. (2018). Effects of Coral, Recycled and Natural Coarse Aggregates on the Mechanical Properties of Concrete. Constr. Build. Mater..

[87] Bui N.K., Satomi T., Takahashi H. (2018). Enhancement of Recycled Aggregate Concrete Properties by a New Treatment Method. GEOMATE J..

[88] Dimitriou G., Savva P., Petrou M.F. (2018). Enhancing Mechanical and Durability Properties of Recycled Aggregate Concrete. Constr. Build. Mater..

[89] Thomas C., Sosa I., Setién J., Polanco J.A., Cimentada A.I. (2014). Evaluation of the Fatigue Behavior of Recycled Aggregate Concrete. J. Clean. Prod..

[90] Pacheco J., De Brito J., Chastre C., Evangelista L. (2019). Experimental Investigation on the Variability of the Main Mechanical Properties of Concrete Produced with Coarse Recycled Concrete Aggregates. Constr. Build. Mater..

[91] Hao Y., Ren Q. (2011). Experimental Research on Mechanical Properties of Recycled Aggregate Concrete. Proceedings of the 2011 International Conference on Multimedia Technology.

[92] Zhao X.Y., Duan M.L. (2013). Experimental Research on Mechanical Properties of Recycled Aggregate Concrete under Uniaxial Loading. Adv. Mater. Res..

[93] Seo T.-S., Lee M.-S. (2015). Experimental Study on Tensile Creep of Coarse Recycled Aggregate Concrete. Int. J. Concr. Struct. Mater..

[94] Sadati S., Khayat K.H. (2016). Field Performance of Concrete Pavement Incorporating Recycled Concrete Aggregate. Constr. Build. Mater..

[95] Kang T.H.-K., Kim W., Kwak Y.-K., Hong S.-G. (2014). Flexural Testing of Reinforced Concrete Beams with Recycled Concrete Aggregates. ACI Struct. J..

[96] Chakradhara Rao M., Bhattacharyya S., Barai S. (2011). Influence of Field Recycled Coarse Aggregate on Properties of Concrete. Mater. Struct..

[97] Kou S.C., Poon C.S., Chan D. (2007). Influence of Fly Ash as Cement Replacement on the Properties of Recycled Aggregate Concrete. J. Mater. Civ. Eng..

[98] Geng Y., Wang Q., Wang Y., Zhang H. (2019). Influence of Service Time of Recycled Coarse Aggregate on the Mechanical Properties of Recycled Aggregate Concrete. Mater. Struct..

[99] Ferreira L., De Brito J., Barra M. (2011). Influence of the Pre-Saturation of Recycled Coarse Concrete Aggregates on Concrete Properties. Mag. Concr. Res..

[100] Pedro D., De Brito J., Evangelista L. (2014). Influence of the Use of Recycled Concrete Aggregates from Different Sources on Structural Concrete. Constr. Build. Mater..

[101] Purushothaman R., Amirthavalli R.R., Karan L. (2015). Influence of Treatment Methods on the Strength and Performance Characteristics of Recycled Aggregate Concrete. J. Mater. Civ. Eng..

[102] Yang K.-H., Chung H.-S., Ashour A. (2008). Influence of Type and Replacement Level of Recycled Aggregates on Concrete Properties. ACI Mater. J..

[103] Letelier V., Ortega J.M., Muñoz P., Tarela E., Moriconi G. (2018). Influence of Waste Brick Powder in the Mechanical Properties of Recycled Aggregate Concrete. Sustainability.

[104] Deng Z., Liu B., Ye B., Xiang P. (2019). Mechanical Behavior and Constitutive Relationship of the Three Types of Recycled Coarse Aggregate Concrete Based on Standard Classification. J. Mater. Cycles Waste Manag..

[105] Bravo M., De Brito J., Pontes J., Evangelista L. (2015). Mechanical Performance of Concrete Made with Aggregates from Construction and Demolition Waste Recycling Plants. J. Clean. Prod..

[106] Letelier V., Ortega J.M., Tarela E., Muñoz P., Henríquez-Jara B.I., Moriconi G. (2018). Mechanical Performance of Eco-Friendly Concretes with Volcanic Powder and Recycled Concrete Aggregates. Sustainability.

[107] Cabral A.E.B., Schalch V., Dal Molin D.C.C., Ribeiro J.L.D. (2010). Mechanical Properties Modeling of Recycled Aggregate Concrete. Constr. Build. Mater..

[108] Kou S.-C., Poon C.-S. (2008). Mechanical Properties of 5-Year-Old Concrete Prepared with Recycled Aggregates Obtained from Three Different Sources. Mag. Concr. Res..

[109] Yang S., Lee H. (2017). Mechanical Properties of Recycled Aggregate Concrete Proportioned with Modified Equivalent Mortar Volume Method for Paving Applications. Constr. Build. Mater..

[110] Huang Y., He X., Wang Q., Sun Y. (2018). Mechanical Properties of Sea Sand Recycled Aggregate Concrete under Axial Compression. Constr. Build. Mater..

[111] Ismail S., Ramli M. (2014). Mechanical Strength and Drying Shrinkage Properties of Concrete Containing Treated Coarse Recycled Concrete Aggregates. Constr. Build. Mater..

[112] Pedro D., De Brito J., Evangelista L. (2015). Performance of Concrete Made with Aggregates Recycled from Precasting Industry Waste: Influence of the Crushing Process. Mater. Struct..

[113] Limbachiya M., Meddah M.S., Ouchagour Y. (2012). Performance of Portland/Silica Fume Cement Concrete Produced with Recycled Concrete Aggregate. ACI Mater. J..

[114] Gómez-Soberón J.M. (2002). Porosity of Recycled Concrete with Substitution of Recycled Concrete Aggregate: An Experimental Study. Cem. Concr. Res..

[115] Vieira J., Correia J., De Brito J. (2011). Post-Fire Residual Mechanical Properties of Concrete Made with Recycled Concrete Coarse Aggregates. Cem. Concr. Res..

[116] Sri Ravindrarajah R., Tam C. (1985). Properties of Concrete Made with Crushed Concrete as Coarse Aggregate. Mag. Concr. Res..

[117] Alengaram U.J., Salam A., Jumaat M.Z., Jaafar F.F., Saad H.B. (2011). Properties of High-Workability Concrete with Recycled Concrete Aggregate. Mater. Res..

[118] Surya M., Vvl K.R., Lakshmy P. (2013). Recycled Aggregate Concrete for Transportation Infrastructure. Procedia-Soc. Behav. Sci..

[119] Folino P., Xargay H. (2014). Recycled Aggregate Concrete–Mechanical Behavior under Uniaxial and Triaxial Compression. Constr. Build. Mater..

[120] Tran D.V.P., Allawi A., Albayati A., Cao T.N., El-Zohairy A., Nguyen Y.T.H. (2021). Recycled Concrete Aggregate for Medium-Quality Structural Concrete. Materials.

[121] Meddah M.S., Al-Harthy A., Ismail A.M. (2020). Recycled Concrete Aggregates and Their Influences on Performances of Low and Normal Strength Concretes. Buildings.

[122] Zega C., Di Maio A. (2006). Recycled Concrete Exposed to High Temperatures. Mag. Concr. Res..

[123] Zega C.J., Di Maio A.A. (2009). Recycled Concrete Made with Different Natural Coarse Aggregates Exposed to High Temperature. Constr. Build. Mater..

[124] Zega C.J., Di Maio A.A. (2011). Recycled Concretes Made with Waste Ready-Mix Concrete as Coarse Aggregate. J. Mater. Civ. Eng..

[125] Zhao H., Liu F., Yang H. (2020). Residual Compressive Response of Concrete Produced with Both Coarse and Fine Recycled Concrete Aggregates after Thermal Exposure. Constr. Build. Mater..

[126] Thomas J., Thaickavil N.N., Wilson P. (2018). Strength and Durability of Concrete Containing Recycled Concrete Aggregates. J. Build. Eng..

[127] Belén G.-F., Fernando M.-A., Diego C.L., Sindy S.-P. (2011). Stress–Strain Relationship in Axial Compression for Concrete Using Recycled Saturated Coarse Aggregate. Constr. Build. Mater..

[128] Corinaldesi V. (2011). Structural Concrete Prepared with Coarse Recycled Concrete Aggregate: From Investigation to Design. Adv. Civ. Eng..

[129] Pedro D., De Brito J., Evangelista L. (2017). Structural Concrete with Simultaneous Incorporation of Fine and Coarse Recycled Concrete Aggregates: Mechanical, Durability and Long-Term Properties. Constr. Build. Mater..

[130] Mohammed N., Sarsam K., Hussien M. (2018). The Influence of Recycled Concrete Aggregate on the Properties of Concrete. MATEC Web Conf..

[131] Gholampour A., Ozbakkaloglu T. (2018). Time-Dependent and Long-Term Mechanical Properties of Concretes Incorporating Different Grades of Coarse Recycled Concrete Aggregates. Eng. Struct..

[132] Butler L.J., West J.S., Tighe S.L. (2014). Towards the Classification of Recycled Concrete Aggregates: Influence of Fundamental Aggregate Properties on Recycled Concrete Performance. J. Sustain. Cem.-Based Mater..

[133] Pani L., Francesconi L. (2014). Ultrasonic Test on Recycled Concrete: Relationship among Ultrasonic Waves Velocity, Compressive Strength and Elastic Modulus. Adv. Mater. Res..

[134] Chen H.-J., Yen T., Chen K.-H. (2003). Use of Building Rubbles as Recycled Aggregates. Cem. Concr. Res..

[135] Xiao J., Zhang K., Akbarnezhad A. (2018). Variability of Stress-Strain Relationship for Recycled Aggregate Concrete under Uniaxial Compression Loading. J. Clean. Prod..

[136] Smiti A. (2020). A Critical Overview of Outlier Detection Methods. Comput. Sci. Rev..

[137] Hodge V., Austin J. (2004). A Survey of Outlier Detection Methodologies. Artif. Intell. Rev..

[138] Chen J., Bao Y. (2025). Effect of Dataset Representation Bias on Generalizability of Machine Learning Models in Predicting Flexural Properties of Ultra-High-Performance Concrete (UHPC) Beams. Eng. Struct..

[139] Gonzalez-Corominas A., Etxeberria M. (2016). Effects of Using Recycled Concrete Aggregates on the Shrinkage of High Performance Concrete. Constr. Build. Mater..

[140] Kou S.C., Poon C.S., Chan D. (2008). Influence of Fly Ash as a Cement Addition on the Hardened Properties of Recycled Aggregate Concrete. Mater. Struct..

[141] Barbudo A., De Brito J., Evangelista L., Bravo M., Agrela F. (2013). Influence of Water-Reducing Admixtures on the Mechanical Performance of Recycled Concrete. J. Clean. Prod..

